# HF-EPR, Raman, UV/VIS Light Spectroscopic, and DFT Studies of the Ribonucleotide Reductase R2 Tyrosyl Radical from Epstein-Barr Virus

**DOI:** 10.1371/journal.pone.0025022

**Published:** 2011-09-27

**Authors:** Ane B. Tomter, Giorgio Zoppellaro, Florian Schmitzberger, Niels H. Andersen, Anne-Laure Barra, Henrik Engman, Pär Nordlund, K. Kristoffer Andersson

**Affiliations:** 1 Department of Molecular Biosciences, University of Oslo, Oslo, Norway; 2 Department of Medical Biochemistry and Biophysics, Karolinska Institute, Stockholm, Sweden; 3 Laboratoire National des Champs Magnétiques Intenses, LNCMI-G, UPR 3228, CNRS, Grenoble, France; University of South Florida College of Medicine, United States of America

## Abstract

Epstein-Barr virus (EBV) belongs to the gamma subfamily of herpes viruses, among the most common pathogenic viruses in humans worldwide. The viral ribonucleotide reductase small subunit (RNR R2) is involved in the biosynthesis of nucleotides, the DNA precursors necessary for viral replication, and is an important drug target for EBV. RNR R2 generates a stable tyrosyl radical required for enzymatic turnover. Here, the electronic and magnetic properties of the tyrosyl radical in EBV R2 have been determined by X-band and high-field/high-frequency electron paramagnetic resonance (EPR) spectroscopy recorded at cryogenic temperatures. The radical exhibits an unusually low g_1_-tensor component at 2.0080, indicative of a positive charge in the vicinity of the radical. Consistent with these EPR results a relatively high C-O stretching frequency associated with the phenoxyl radical (at 1508 cm^−1^) is observed with resonance Raman spectroscopy. In contrast to mouse R2, EBV R2 does not show a deuterium shift in the resonance Raman spectra. Thus, the presence of a water molecule as a hydrogen bond donor moiety could not be identified unequivocally. Theoretical simulations showed that a water molecule placed at a distance of 2.6 Å from the tyrosyl-oxygen does not result in a detectable deuterium shift in the calculated Raman spectra. UV/VIS light spectroscopic studies with metal chelators and tyrosyl radical scavengers are consistent with a more accessible dimetal binding/radical site and a lower affinity for Fe^2+^ in EBV R2 than in *Escherichia coli* R2. Comparison with previous studies of RNR R2s from mouse, bacteria, and herpes viruses, demonstrates that finely tuned electronic properties of the radical exist within the same RNR R2 Ia class.

## Introduction

**Figure 1:**
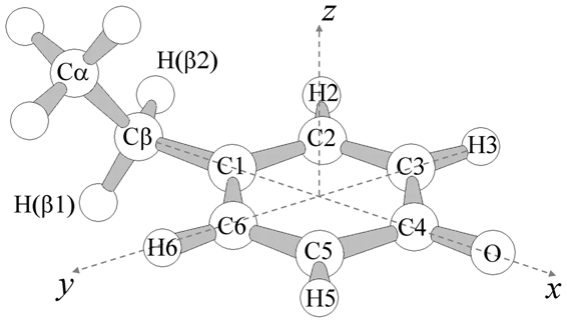
The tyrosyl radical. Molecular sketch of a tyrosyl radical with the definition of the dihedral angle θ used to describe the orientation of the α-carbon (C6-C1-Cβ-Cα). An angle of θ = 0° corresponds to placing the α-carbon in the plane of the phenoxyl ring (xy), and θ = 90° corresponds to an orientation perpendicular to the plane of the ring.

Ribonucleotide reductase catalyzes the conversion of ribonucleotides to the corresponding deoxyribonucleotides in all living organisms *via* a radical-based chemical mechanism, thereby providing and controlling the pool of precursors necessary for DNA synthesis and repair [1], [2], [3], [4]. Based on differences in the generation of the free radical, the amino acid sequence, and the overall quaternary structure [1], RNRs can be grouped into three different classes (I, II, and III). Class I RNR, the most common class, found in almost all eukaryotic organisms is oxygen dependent and consists of two subunits, designated R1 and R2 [2]. These can assemble into enzymatically active homodimeric tetramers (R1_2_R2_2_) and higher order oligomers [5], [6], [7]. Most R2s generate a stable tyrosyl radical in the vicinity of a dimetal-oxygen cluster [3], [6]. This radical transfers from the radical dimetal site in R2, over a distance of ca. 35 Å, to the active site of the R1 subunit, where it forms a thiyl radical. Its transfer likely is facilitated by a conserved array of hydrogen bonded amino acids [2], [8], [9]. Herpes viruses are ubiquitous eukaryotic pathogens infecting a large variety of animal species. They share similar architecture with a double-stranded DNA genome encased within a proteinaceous cage. Many herpes viruses, including those of the α-and γ-subfamilies, encode for an active RNR in their genomes. Most likely, the viral DNA replication relies on *de novo* synthesis of deoxyribonucleotides by the viral RNR under conditions in which the host cell RNR is inactive, such as in non-dividing cells [10]. Herpes viral R2 subunits have limited similarity in amino acid sequence compared with mammalian and bacterial homologues. Yet, previous studies of the RNR form herpes simplex virus (HSV) 1 and 2 indicated a similar catalytic mechanism as in *Escherichia coli* class Ia RNR [11], [12]. Epstein-Barr virus (EBV) is classified in the gamma subfamily of herpes viruses and represents one of the most common pathogenic viruses in humans worldwide. EBV R2 has low sequence similarity with mouse R2 (27.0% sequence identity and 53.7% sequence similarity (as calculated with the blosum62 matrix ), human p53R2 (26.7% and 52.7%), herpes simplex virus (HSV) 1 (36.7% and 59.6%), HSV 2 (36.4% and 60.9%) and *E. coli* R2 (27.0% and 53.7%). Amino acid sequence alignments indicate that the equivalent amino acid for the conserved metal coordinating aspartate in most R2s is a glutamate (Glu61 in EBV R2). The virus may induce development of several diseases such as infectious mononucleosis, and is associated with neoplasms, including lymphomas and carcinomas [13]. In this context, the viral RNR is an important drug target for EBV [14], [15]. Certain small organic molecules are potential inhibitors of herpes viral RNRs and thus can be employed for the treatment of herpes viral infections by targeting the R2 tyrosyl radical site. For example, hydroxyurea (HU), a potent reductant, diminishes the EBV genome in Burkitt's lymphoma cell lines *in vitro*, whereas prolonged exposure may lead to HU resistance in some cell lines [16]. Generally, an understanding of the specificity of inhibitors requires detailed knowledge of the accessibility, reactivity and the electronic and magnetic characteristics of the radical site [17]. The tyrosyl radical from several different R2s have been characterized, showing that the magnetic properties of the radical unit are strongly correlated with the dihedral angles of its tyrosyl β-methylene protons [18], [19], [20], [21] (Figure 1). Applying high-field/high-frequency electron paramagnetic resonance (HF-EPR) allows measurement of the *g*-tensor anisotropy of the radical center with high accuracy. Previous studies of tyrosyl radicals demonstrated that the *g*-values are sensitive probes for the local electrostatic environment. HF-EPR measurements showed that there are clear differences in the *g*-tensor anisotropy between different RNR R2s from class Ia [22], [23], [24]. Such differences are assumed to arise from variations in the hydrogen bonding interaction with a nearby hydrogen for both the mouse R2 radical and the Y_D_ radical from photo system II (PS II) [22]. In this work the physicochemical properties of the tyrosyl radical from EBV R2 were characterized experimentally and theoretically with the use of X-band, HF-EPR, resonance Raman (rRaman) spectroscopy and density functional theory (DFT). Further, we report ultraviolet/visible light spectroscopic (UV/VIS) experiments probing the dissociation of iron – in different oxidation states – and the radical with metal chelators and radical scavengers, respectively. We compare our results with previous observations for RNR R2s from mouse, bacteria, and other herpes viruses and discuss possible implications.

## Results and Discussion

### Low temperature EPR spectra of the EBV R2 tyrosyl radical at two different microwave frequencies

The free tyrosyl radical from metal-free EBV R2 reconstituted with Fe^2+^ has been studied in the temperature range *T* = 3–30 K at two EPR microwave frequencies, 9.6 (X-band) and 285 GHz (high-frequency/field, HF-EPR). The formation of the tyrosyl radical in EBV R2 occurs with ∼1 radical per R2 dimer, which is similar to our earlier studies on human and mouse p53R2 and mouse R2 under similar reconstitution conditions. These values are higher than those obtained for HSV 1 and HSV 2 R2, which were calculated to be 0.3–0.6 radical per R2 dimer [21], [25], [26], [27], [28], [29].

**Figure 2:**
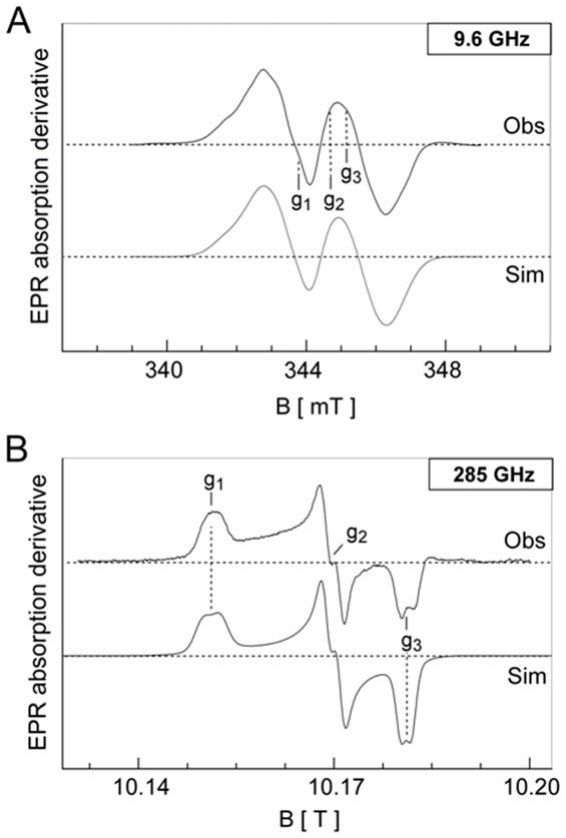
X-band and HF-EPR spectra of EBV R2. Low-temperature EPR spectra of the tyrosyl radical of EBV R2. A protein concentration of ∼140–150 µM was used for these measurements. (**A**) The upper spectrum (Obs) shows the 9.6 GHz (X-band) resonance envelope recorded at T = 26 K with a microwave power of 0.08 mW and modulation amplitude 0.4 mT, and the lower spectrum shows its computer simulation (Sim) (**B**) The upper spectrum (obs) shows the 285 GHz resonance envelope recorded at T = 15 K with a modulation amplitude of 0.4 mT and microwave power in the µW range, and the lower spectrum its computer simulation (Sim). The derived spin-Hamiltonian parameters are listed in Table 1.

**Figure 3:**
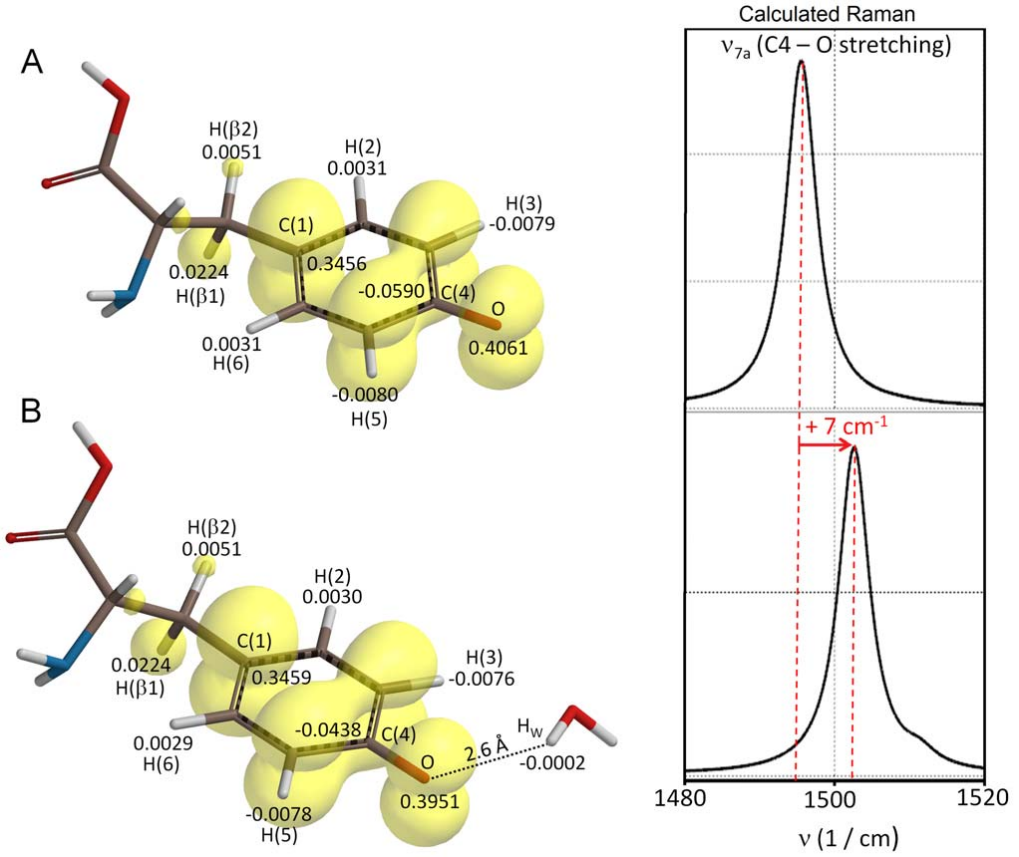
Spin density distribution on the tyrosyl radical in EBV. The yellow spin spin density surfaces of the tyrosyl radical obtained by density functional theory (DFT/UB3LYP/6-311++G(d,p) in gas phase (0.0015 au isovalue), for the neutral forms after geometry optimization (the dihedral angle θ was constrained to 30°) in the absence (A) and in the presence (B) of a water molecule located close to the phenoxyl oxygen. The isosurfaces show the larger positive spin density on H(β1) with respect to the H(β2) proton. The numerical values (au) derived from Natural Atomic Populations (NAP) analyses are also given for the H(3,5) and H(2,4) protons as well as for the C4 and the tyrosyl oxygen atoms. This level of theory gives a calculated C(4)-O bond length of (A) 1.252 Å and (B) 1.256 Å. The theoretically derived Raman spectra (DFT/UB3LYP/6-311++G(d,p), 3 cm^−1^ Lorentzian line band-width) are shown on the right side of the panel, and highlight the frequency shift associated to the C(4)-O stretching vibration (ν_7a_, Wilson notation) when the phenoxyl oxygen interacts with a water molecule.

The observed first-derivative EPR spectra are shown in Figure 2A and 2B, respectively. The X-band EPR envelope of EBV R2 (Figure 2A, Obs) is similar to the spectra observed for HSV 1 and mouse R2 as well as for HSV 2 R2 [12], [23], [24], [27], [29], [30]. As expected, the anisotropic *g*-tensor components are poorly resolved under this microwave frequency. The HF-EPR (285 GHz) spectrum and *g*_iso_ from X-band on the other hand clearly show the *g*-tensor anisotropy with values at *g*_1_ = 2.0080, *g*_2_ = 2.0043, and *g*_3_ = 2.0021 (Figure 2B, Obs). The spin concentration of the tyrosyl radical was determined as one spin equivalent per dimer, and remained constant in the temperature range examined. The observed total resonance width (Δ*B*∼6.5 mT) is characterized by a composite resonance line, due to the presence of several anisotropic hyperfine splitting components (A), in analogy with previous analyses of the mouse and HSV 1 R2 spectra at 9.6 GHz. This phenomenon arises from the interaction of the unpaired spin on the phenolate with magnetically different hydrogen nuclei of the radical carrying tyrosine backbone as well as the β_1,2_-H protons (Figure 1 and Figure 3). The EPR signal line-shape and resonance features depend on the rotational configuration of the tyrosyl ring. Therefore, spectra simulations that fit both the X-band and high-field EPR envelopes (Figure 2A and 3B, Sim), followed by comparison with values from the literature, allowed an estimate for the rotational angle θ of ∼30° (see Scheme 1 for the definition of the θ rotational angle and the DFT generated spin density at UB3LYP/6-311++G** of the radical model in Figure 3). According to previous theoretical DFT studies on model tyrosyl radical systems such an angle falls at a local minimum in the potential energy curve as described by the rotational motion of the phenoxyl plane with respect to the α,β-carbons [18]. The simulation parameters used are collected in Table 1, together with previously determined *g*, A and θ values for the tyrosyl radical from *E. coli* R2 [23], [31], mouse R2 [23], [24], [27], [32], [33], HSV 1 R2 [23], [24], [27], [32], *Salmonella typhimurium* R2 [34] and PS II Y_D_ [22], [34] included for comparison. Previous work demonstrated that measurement of the *g*-value anisotropy can be used as a probe for the presence of a hydrogen bond to the phenol oxygen of the tyrosyl radical. The absence of a hydrogen bond, as occurring for the tyrosyl radical in *E. coli* R2, results in a large *g*_1_-value of ca. 2.0090 [22], [23], [31]. In contrast, when a positive charge is located in close vicinity such that a hydrogen bond is formed with the tyrosyl phenol oxygen, the spin density on the phenolic moiety is lowered proportionally (Figure 3B), resulting in smaller *g*_1_-values [18], [20], [22], [23], [32], [33], [35], [36]. In the crystal structure of the diferric (Fe^3+^-O^2−^-Fe^3+^) form of mouse R2 without a tyrosyl radical, two water molecules are present in close proximity of the tyrosyl radical site [37]. Furthermore, in the Y107^•^-radical site of the Y122F/W107Y double mutant from *E. coli* RNR R2, the radical forms a strong H-bond and displays a low *g*_1_-value (*g*_1_ = 2.0069 [23]). In the latter case, one of the water molecules in the crystal structures is located substantially closer to the tyrosyl radical than found in mouse R2 [23], [38]. The *g*_1_-value of p53R2 [39] has not been reported, but is indicated in chapter 1 in reference ([2]) to have a low *g*_1_-value (it is possible that the diiron center shows a mixed valence signal). Therefore, the perturbation of the *g*-tensor values of the tyrosyl radical in R2s usually originates from the local electrostatic environment [40]. The energies of the excited states associated with the radical unit, which are characterized by the nonbonding molecular orbitals of the phenoxyl oxygen, can be modified in a way that spin-orbit mixing occurs between low-lying excited states with the ground state, leading to alterations in the *g*-anisotropy. The sensitivity of the *g*_1_-values for the tyrosyl radical to the local chemical environment must also be reflected in electrochromic shifts in infrared (IR), Raman (see Figure 3) and rRaman spectra. The observed *g*_1_-value of the tyrosyl radical in EBV R2 is lower than that of *E. coli* R2, but slightly higher than of both mouse R2 and HSV 1 R2. Therefore, in EBV R2 spin-mixing and a hydrogen bond to the tyrosyl radical can be present. If a hydrogen bond is present, the strength of the latter must be weaker than those observed in mouse and HSV 1 R2. However, the molecular basis of such an interaction cannot be determined by simple continuous-wave EPR experiments alone (*vide infra*). Davies D-electron nuclear double resonance (ENDOR) studies of mouse and HSV 1 R2 demonstrated that, while both display identical *g*_1_ values, the distance (*d*) between the exchangeable H/D (probably originating from a water molecule) is slightly shorter in HSV 1 R2 (*d* = 1.86 Å) compared with mouse R2 (*d* = 1.89 Å) [27]. Thus, if a similar interaction in EBV R2 were present, the expected distance to the hydrogen should be slightly larger (1.9≤*d*≤2.6 Å). Other moieties, than an exchangeable hydrogen from a water or hydroxide molecule, such as amino acids sidechains from the protein located in proximity of the tyrosyl radical site could contribute to the decrease of the *g*_1_-component. As found for example in PS II Y_D_ (D2-Tyr160 from the cyanobacterium *Synechocystis*), the low *g*_1_-value of the tyrosyl radical arises from positive charges located close to the phenol-oxygen (see Table 1). In this case, the hydrogen bonding interaction is not formed with a water or hydroxide molecule, but with a neighbouring histidine residue. Its imidazole moiety can serve as an acceptor for the phenolic proton upon oxidation, forming a hydrogen bond to the phenolic oxygen of the neutral tyrosyl radical as demonstrated by combined mutagenesis studies and Mims electron spin echo-ENDOR measurements [41]. Thus, any shift of the *g*_1_-tensor component derived from HF-EPR measurements must be analyzed with caution as an indication for the presence of a water or hydroxide molecule in close proximity of the tyrosyl radical.

**Table 1:** The *g*–tensor, *hf*–tensors (A, mT), line–width tensor (LW, mT), and the rotational θ angle of the α-carbon (°, deg) for the tyrosyl radical in Class Ia, Class Ib RNRs and the Y_D_ radical in photosystem II.

EPR-tensors	EBV R2	HSV1 R2	*E. coli* R2	Mouse R2	*S. typhimurium* R2	PS II Y_D_
*g*_1_	2.0080	2.0076	2.0090	2.0076	2.0089	2.0076
*g*_2_	2.0043	2.0043	2.0046	2.0043	2.0043	2.0043
*g*_3_	2.0021	2.0022	2.0022	2.0022	2.0021	2.0022
H_3,5_ A_xx_	−0.80/−0.70*	−0.28/−0.43*	−0.96	−0.91/−0.73*	−1.15	−0.91
H_3,5_ A_yy_	−0.50/−0.40*	−0.28/−0.18*	−0.28	−0.44/−0.48*	−0.25	−0.26
H_3,5_ A_zz_	−0.30/−0.20*	−0.47−/0.64*	−0.70	−0.66/−0.58*	−0.71	−0.70
H_β1_ A_xx_	2.60	2.28	1.96	2.14	1.05	0.72
H_β1_ A_yy_	1.80	2.07	2.12	1.90	0.74	1.05
H_β1_ A_zz_	1.40	1.78	1.96	2.15	0.95	0.72
H_β2_ A_xx_	0.40	0.39	0.18	0.95	<0.3	0.19
H_β2_ A_yy_	0.30	0.25	−0.07	0.25	<0.3	0.19
H_β2_ A_zz_	0.30	0.28	−0.07	0.57	<0.3	0.51
LWx	0.38	0.33	-	0.45	0.58	-
LWy	1.0	0.33	-	0.35	0.53	-
LWz	1.1	0.33	-	0.44	0.37	-
θ	30	35	30	35	75	82
Ref.	This work#	[27], [33]	[31], [40], [65]	[33]	[34]	[22], [34]

*Non-equivalent H_3_ and H_5_ protons.

# The proton (H) *hfc* signs were derived from the DFT analyses. The definition of the torsional angle θ was given in Scheme 1.

**Figure 4:**
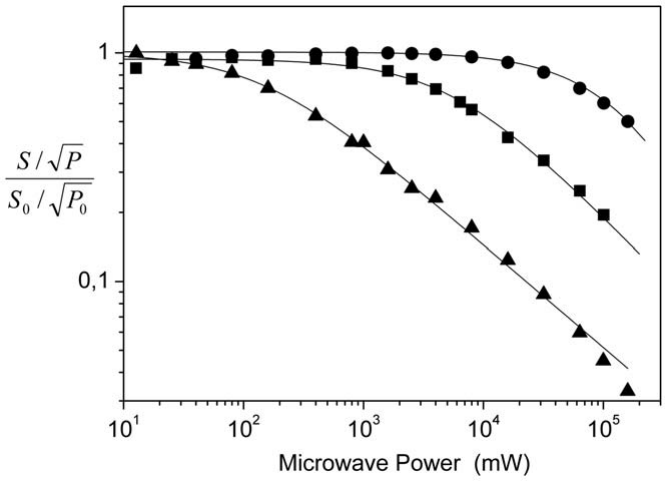
EPR microwave power saturation. The microwave power saturation curves for EBV R2 as derived from X-Band EPR measurements performed at *T* = 26 K (▴), *T* = 50 K (▪), and *T* = 100 K (•). The *b*-values were equal to 1 for *T* = 26 K and 50 K and <1 for *T* = 100 K. P_1/2_ (26 K) = 0.14 mW, P_1/2_ (50 K) = 5 mW, P_1/2_ (100 K)≥150 mW. *S*: double integrated signal amplitude, *P*: microwave power, *S*_0_ and *P*_0_ are reference values obtained at non-saturating conditions.

EPR relaxation measurements allow closer investigation of the chemical environment of the tyrosyl radical through observation of the progressive microwave power saturation properties of the EPR spectra in the temperature range of *T* = 26–100 K (Figure 4). When an effective magnetic coupling occurs between the radical and the diferric metal-oxygen cluster, the radical relaxation properties, as indexed by the half-saturation value (*P*_1/2_), become larger than the one observed in the absence of magnetic coupling. Compared with previous studies carried out on mouse and HSV 1 R2 (with values of *P*_1/2_ = 1.2 mW and *P*_1/2_ = 3 mW at 30 K, respectively), the resonance saturation for the EBV R2 radical occurs at a lower applied microwave power (*P*_1/2_ = 0.14 mW), but higher than for *E. coli* R2 (*P*_1/2_ = 0.05 mW) [30], [42]. Thus, the magnetic interaction between the iron-oxygen cluster and the tyrosyl radical must be weaker than that estimated for both mouse and HSV 1 R2. The presence of a glutamate in EBV R2 (Glu61) in the amino acid sequence position of the common iron-coordinating aspartate in HSV 1 and mouse R2, may have an impact on these observed differences. Similar to *E. coli* R2 and *S. typhimurium* R2, the *P*_1/2_ value in EBV R2 derived here is larger than that observed for a free tyrosyl radical lacking any magnetic interaction with another paramagnetic center [42]. For EBV R2 we did not succeed to trap the mixed valence (Fe^2+^Fe^3+^) complex or to detect integer spin signals, (with *g* values in the range 8≤*g*≤16) of the diferrous site with the chemical mediators employed in this study. This electronic characteristic in EBV R2 distinguishes it from those previously observed by us for mouse R2, where both signals can be detected, and from HSV 1 R2, which exhibit the mixed valence signal under mildly reducing conditions [43], [44]. The mouse R2 integer spin EPR signal can be explained by the relatively small zero field splitting parameters designated Δ or δ, making it an observable signal for X-band EPR [9], [45]. Thus, there are clear differences in the electronic characteristics of the tyrosyl radical site in EBV R2 compared with previously studied R2s [21], [27].

### Resonance Raman spectroscopic studies of the EBV R2 tyrosyl radical

**Figure 5:**
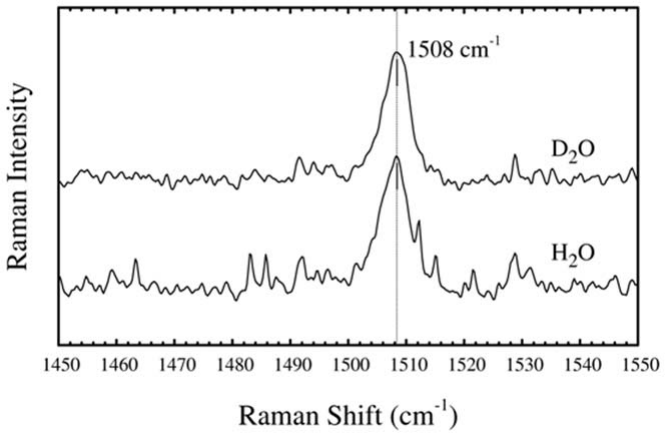
Resonance Raman spectra. The resonance Raman spectra of the tyrosyl radical in EBV R2 and the effect of D_2_O exchange recorded with the following settings: *λ*_exc_ = 410 nm, laser power at sample ∼5 mW, 60 scan, 60 sec/scan, *T* = 77 K.

In order to investigate in more detail the basis for the low *g*_1_-value, rRaman spectroscopy was employed. The rRaman and IR spectroscopy are excellent complementary techniques to HF-EPR spectroscopy, because even weak interactions between the tyrosyl radical and molecules in its close proximity can be detected. Tyrosyl radicals show a characteristic light absorption maximum at ca. 410 nm (see below) that is usually used for excitation in rRaman spectra of the radical. Vibrational spectroscopy can identify redox-linked structural changes associated with electron transfer reactions, as shown for the tyrosyl radical (Y122^•^) in *E. coli* RNR. For the latter such an electron transfer reaction has been proven to be coupled to a conformational change in the R2 structure [46]. One vibrational characteristic of the radical, the phenoxyl ν_7a_ mode (Wilson notation) with components of the C4-O stretching vibrations (Figure 5), is a sensitive marker for hydrogen bonds in this context. This vibrational mode is strongly enhanced when rRaman is employed at excitation frequencies *ν*_ex_ around 405–415 nm, i.e. very close to the light absorption maximum of the tyrosyl radical. Without a nearby hydrogen bonding interaction, the ν_7a_ vibration of the tyrosyl radical has been observed between 1497 and 1501 cm^−1^ in *E. coli* R2, whereas in mouse R2 it is observed at 1515 cm^−1^ [47]. In mouse R2 a water molecule in close proximity (-O_phenol_…H distance of 1.89 Å) with exchangeable hydrogens is present as demonstrated with pulsed ENDOR [27]. As shown in Figure 5 (lower line), the EBV R2 tyrosyl displays a C4-O stretching vibration at slightly higher energy than that observed in *E. coli* R2 with the *ν*_7a_ vibration maximum at 1508 cm^−1^, which is at lower energy than that observed for mouse R2. Therefore, our rRaman results are consistent with our HF-EPR data on EBV R2, with the tyrosyl radical displaying a *g*_1_-value in between those observed for mouse and in *E. coli* R2. In EBV R2 the observation of such an intermediate frequency mode is, in principle, supportive of the existence of a hydrogen bonded tyrosyl radical with a water molecule as hydrogen bond donor. If present, such a hydrogen bond is weaker than in mouse R2.

### Theoretical analyses of the influence of a hydrogen-bond to a water molecule on the tyrosyl radical mode frequency and EPR spectra

**Figure 6:**
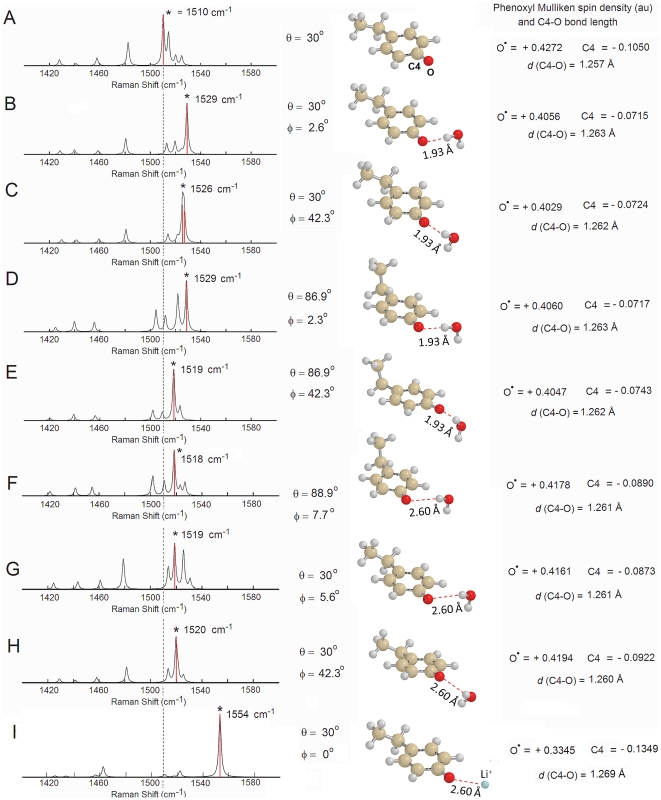
Theoretical resonance Raman modes. Calculated Raman spectra of the tyrosyl radical model system (ethylphenoxyl radical, neutral form) in the absence (**A**) or presence (panel **B–H**) of a water molecule in close vicinity (**B**, **C**, **D**, and **E**, *d* = 1.93 Å) or placed further away (**F**–**H**, *d* = 2.60 Å) from the phenoxyl oxygen. Panel **I** shows the interaction with nearby positive charge (Li^+^). The calculations were performed by density functional theory (DFT/UB3LYP/6-31G(d,p)) in gas phase. No frequency scaling was applied and a 3 cm^−1^ band width (Lorentzian-line) was used. The angle φ is defined as the angle (°, deg) formed between the phenoxyl plane and the C4_phen_-O_phen_….H_water_ plane. The definition of the angle θ is given in Figure 1. The numerical values for the Mulliken spin density calculated for the phenoxyl (-O^•^) oxygen (positive) and the C4 atom (negative) are shown on the right-hand side of each panel. The minor bond elongation observed in the calculated C4-O bond length upon interaction of the phenoxyl oxygen with the water molecule is also shown. The asterisk (*) in each panel indicates the position of the ν_7a_ mode. The thin dashed vertical line serves as a visual marker for the unperturbed tyrosyl radical to guide to the eyes.

In order to test the impact of a water molecule in the proximity of the tyrosyl radical on the Raman spectra, DFT calculations were performed using a simplified model, the *p*-ethylphenoxyl radical. This has been demonstrated to be a good model system before [36]. The molecular backbone of the radical unit in EBV R2 is characterized by a rotational configuration of the tyrosyl ring θ constraint at 30°, as derived our EPR results. Such analyses gives an estimation of the C4-O stretching vibration trends (B3LYP/6-31G(d,p)) either in the presence or absence of a hydrogen bonded water molecule. The calculated Raman spectra are shown in Figure 6 (without enhanced resonance effects) together with the optimized structures with and without a water molecule placed close to the tyrosyl group at a distance slightly longer (1.93 Å) than that spectroscopically observed in mouse R2 (Figure 6, panel B–E). The calculated C4-O stretching vibration in the absence of a hydrogen bonded water molecule occurs at 1510 cm^−1^ (Figure 6A), a value identical to that obtained for the tyrosyl radical by Johnson and coworkers [48]. The calculated ν_7a_ vibration in this simplified model closely agrees with the Fourier transform-IR analyses on the tyrosinate and tyrosyl radical by Barry and coworkers (tyrosyl radical, ν_7a_ = 1516 cm^−1^), who have explicitly considered the effect of an amino acid backbone in their theoretical calculations (UB3LYP/6-31++G(d,p) as well as from our calculations using the entire tyrosyl radical backbone, computed at a higher level of theory (UB3LYP/6-311++G(d,p), ν_7a_ = 1496 cm−1, Figure 3)) [49]. The value does not differ substantially from results obtained using the *p*-methoxyphenoxy radical as a model system, which employed a different level of theory (BPW91/6-31G**, ν_7a_ = 1496 cm^−1^) [50]. When a water molecule is placed at a distance of 1.93 Å to the phenoxyl plane (and a φ = 2.6° after optimization), the ν_7a_ vibration shifts significantly to longer wave number at 1529 cm^−1^ (Figure 6B) but slightly decreases to 1526 cm^−1^ (Figure 6C) if a water molecule is placed outside of the phenoxyl plane (φ∼42° after optimization). When the rotational configuration of the tyrosyl ring is lifted from the constraint of 30°, this angle after optimization, refined to slightly less than 90° (θ = 89.1°) [18]. Here a water molecule placed in the phenoxyl plane at the same distance of 1.93 Å, gives for the ν_7a_ vibration a similar shift at 1529 cm^−1^ (Figure 6D) but it exhibits a larger decrease to 1519 cm^−1^ (Figure 6E) when is moved out of the phenoxyl plane (φ∼42° after optimization). Upon placement of the water molecule further away (2.60 Å) (Figure 6F and 6G) an effective perturbation of the ν_7a_ vibration is reduced proportionally (ν_7a_ = 1518 cm^−1^ and 1519 cm^−1^, respectively). This is accompanied by an increase of the phenoxyl (-O•) Mulliken spin density and Δν = 8–9 cm^−1^ together with a decrease in the C4-O bond length. Clearly, both properties tend to approach the structural and electronic parameters calculated without nearby water. By placing a positive charge (Li^+^) close to the phenoxyl oxygen (O^•^-Li^+^ = 2.60 Å, on the phenoxyl plane, φ∼0°) the perturbation of the ν_7a_ vibration becomes nonetheless very large (Figure 6I, ν_7a_ = 1554 cm^−1^) (see also Information S1). The C4-O stretching frequency (ν_7a_ vibration) is characterized experimentally by a narrow bandwidth, of approximately 5 cm^−1^, as shown in Figure 5. This vibration is thus expected to be influenced by i) the relative angle of the vector between a hydrogen bonding neighboring molecule and the phenol oxygen and the phenol plane, ii) the distance of the hydrogen bonded molecule to the phenol oxygen and iii) by the conformational orientation of the phenoxyl group with respect to the protein backbone, represented here by a simple ethyl molecule. The impact of molecular conformation on the calculated g-tensor and hyperfine tensor (A_H_) components complemented by their further modulation due to dielectric effects associated to the medium have been discussed thoroughly in other reports, and thus are not further addressed here [35], [36], [50], [51], [52].

### Basis for the lack of deuterium shift in EBV R2 rRaman spectra

In order to probe the presence of a water molecule with exchangeable hydrogens located in proximity of the tyrosyl radical in EBV R2, we experimentally investigated the frequency shifts of the tyrosyl C4-O rRaman stretching vibration with deuterated EBV R2. In mouse R2, deuterium exchange of the protein clearly results in a 5 cm^−1^ shift to a lower energy of 1510 cm^−1^ compared with spectra of the non-deuterated protein. This effect was not observed in *E. coli* R2 [47]. In the former, the exchangeable hydrogens from a water molecule are located in proximity of to the phenol plane as detected by ENDOR. Interestingly, in EBV R2 a similar deuterium induced shift in the rRaman spectrum was not observed (Figure 5, upper line). Yet, this result does not preclude the presence of a water molecule as a hydrogen bond donor in close vicinity of the tyrosyl radical for the following reasons: i) Assuming a buried location of the water in the protein interior, its hydrogens may not be readily exchangeable to deuterium; ii) The distance from an immobilized water molecule to the tyrosyl residue could be too large to be detectable in our rRaman measurements. This scenario would be consistent with the theoretical Raman frequency shift calculated (Figure 6G) where a water molecule is placed close to the phenoxyl plane at a distance of 2.60 Å from the radical site. In this case, any deuterium shift is expected to be extremely small (<2 cm^−1^); iii) The absence of a deuterium shift could also arise from interactions with a positive charge located close (2.60 Å) to the tyrosyl radical site. However, the theoretically calculated effects on the phenoxyl oxygen spin-density and the ν_7a_ vibration, using a Li^+^ placed at 2.60 Å from the phenoxyl oxygen mimicking the presence of such a charged group nearby, are too large (ν_7a_ = 1554 cm^−1^, Figure 6I). Thus, the latter scenario seems unlikely; iv). The most probable explanation is a water molecule as a hydrogen bond donor in EBV R2 with similar overall structural relations to the tyrosyl radical as in HSV 1 R2. This is characterized by a distance of the water to the tyrosyl radical of >2.5 Å together with a large angle between the phenoxyl plane and the C4-O_phen_… H_water_ plane (φ) (Figure 6H, calculated ν_7a_ = 1520 cm^−1^ and hydrogen bond C4-O…H distance of 2.60 Å). This structural organization would suitably explain the lack of an observable deuterium shift for EBV R2.

### Preliminary observations of interaction of EBV R2 with radical scavenger and metal-ion chelators probed by UV/VIS spectroscopy

**Figure 7:**
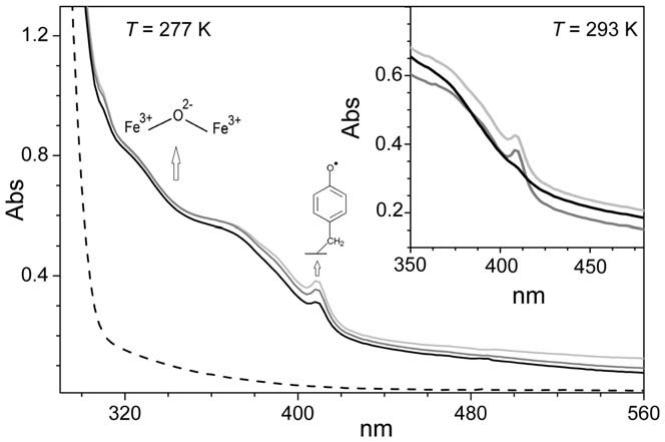
UV-VIS light absorption spectra of tyrosyl radical iron cluster formation and reduction. Light absorption spectra of metal-free EBV R2 before (dashed-line) and after reconstitution of the diferric (Fe^3+^–O^2−^–Fe^3+^) metal site (light-grey line). Representative spectrum obtained under anaerobic conditions for the incubation of reconstituted EBV R2 with 4 mM HU recorded after 200 s (dark grey line) and after 20 min (black line), *T* = 277 K. The inset shows the resulting spectra obtained at higher temperature (*T* = 293 K), before (light-grey line) and after anaerobic incubation with HU (200 s, dark grey line; 400 s, black line). The protein concentration was ∼130 µM in a pH 7.5, 50 mM HEPES buffer.

Certain radical scavengers, such as HU (used since long time in clinical treatment of some cancers), function as potent inhibitors of RNR activity and are attractive drug candidates [2] [53]. HU reduces the tyrosyl radical and large differences in reactivity of HU have been documented between *E. coli* R2 and both mouse R2 and HSV 2 R2s. The tyrosyl radicals of mouse R2 and HSV 2 R2 are more reactive and are scavenged substantially faster than that of *E. coli* R2. The radical quenching process occurs also more rapidly with some hydrophobic radical scavengers, such as *p*-alkoxyphenol compounds, than with HU [28], [54], [55]. Upon reconstitution of the dimetal site of metal-free EBV R2 with Fe^2+^ and exposure to air, relatively strong and characteristic light absorption bands are formed with distinctive absorption features for the diferric center and the tyrosyl radical are apparent at ∼320, 360 nm and ∼410 nm, respectively (Figure 7, light grey line). The strong 320–360 nm band originates from oxo-to-Fe^3+^ dimer charged transfer (CT) transitions. When the diferric active EBV R2 protein was incubated under aerobic conditions with HU, quenching of the tyrosyl radical signal occurred slowly at *T* = 277 K with the total radical quenching occurring after ∼25 min. The quenching rate however was substantially increased at higher temperature (*T* = 293 K), and the absorption signal associated with the tyrosyl radical disappeared after 7 min.

**Figure 8:**
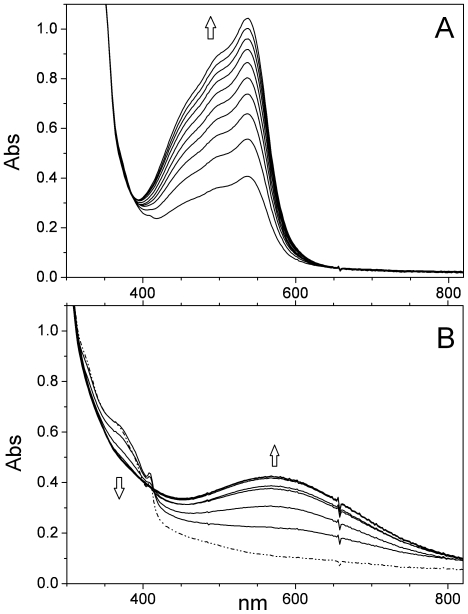
Reaction of reconstituted EBV R2 with scavengers. (**A**) Representative light absorption spectra recorded at *T* = 288 K of reconstituted EBV R2 (∼130 µM, pH 7.5, 50 mM HEPES buffer) after addition of 1 mM bathophenanthrolinesulphonate and 4 mM HU, recorded in 10 min intervals under anaerobic conditions. (**B**) Reconstituted EBV R2 (∼130 µM) incubated with 1 mM catechol at pH 7.5 under anaerobic conditions at *T* = 293 K. The light absorption spectra were recorded at 30 s intervals. The dashed-dotted line represents the spectrum of the reconstituted diferric protein without any metal-ion chelator.

In mouse R2 the reaction of the diferric center with HU leads to reduction of Fe^3+^ followed by rapid dissociation of Fe^2+^ from the protein [56]. For EBV R2, such an effect does not appear prominent (Figure 8). Thus, the reductive ability of HU towards the diferric center in EBV R2 is at an intermediate level between those observed for mouse R2 (very fast) and *E. coli* R2 [55] (non effective).

In order to obtain preliminary knowledge of the dynamics of this reaction in EBV R2, the reconstituted active tyrosyl-radical containing ferric protein was incubated under anaerobic conditions in the presence of HU and bathophenanthrolinesulphonate, a potent Fe^2+^ chelator. The Fe^2+^ complexation by bathophenanthrolinesulphonate was monitored following formation of an absorption band at *λ*_max_ at 535 nm, as described previously [56], [57]. The complex formation process of Fe^2+^ occurred more slowly (Figure 8A) and in parallel with a decrease in the absorption band at 410 nm, which is associated with tyrosyl radical quenching. In order to examine this further an analogous experiment with HU and desferrioxamine, which chelates with Fe^3+^ with formation of a typical light absorption at ∼430 nm (53) was carried out. In contrast to mouse R2, the reaction with EBV R2 did not result in this characteristic absorption after 3 hrs (data not shown). From these preliminary results we conclude that iron dissociated from EBV R2 in the presence of HU is predominantly in the Fe^2+^ state. This implies that HU reacts with both the radical and Fe^3+^ at the diiron site in the protein, as probably also in mouse R2; and that Fe^2+^ is more weakly bound than Fe^3+^ in the dimetal site (Table 2) [54], [58].

**Table 2:** Summary of the UV/VIS spectroscopic experiments on EBV R2 in comparison with similar studies performed on other R2s.

	Bathophenanthroline-sulphonate	Catechol
EBV R2	+	++
*E. coli* R2 [56]	−	−
Mouse R2 [55], [56]	+++	+++
HSV2 R2 [28]	n/a	++

The extent of the reactions was evaluated semi-quantitatively. The symbols indicate (+) positive reaction and (−) no reaction.

The mobilization of Fe^3+^ from EBV R2 by catechol, both a potent metal chelator and radical scavenger [28], was followed spectrophotometrically. The smaller chatecholate compound is a more effective Fe^3+^ chelator than the larger desferrioxamine, as it reacts without any addition of HU with EBV R2. The reaction resulted in a rapid decline of the absorbance at both 360 nm and 410 nm (Figure 8B) together with formation of a blue-violet catecholate to Fe^3+^ CT transitions around 600 nm. Thus, in these experiments with EBV R2, scavenging of the tyrosyl radical occurs concomitant with dissociation of the diferric center, similar to the analysis for HSV 2 R2 [28]. In contrast to *E. coli* R2, in EBV R2 catechol seems to react with the diferric center. The data imply that the reactivity of the tyrosyl radical with catechol in EBV R2 is substantially higher than for *E. coli* R2 and very similar to that of HSV 2 R2 (Table 2).

### Conclusion

Through a combination of EPR, HF-EPR, UV/VIS and rRaman spectroscopy the electromagnetic characteristics of a spectroscopically unique tyrosyl radical from EBV R2 have been determined. The *g*_1_-value from EPR and HF-EPR, and the rRaman shift indicate that the tyrosyl radical is hydrogen bonded, yet deuterium exchange of the protein had no effect on the rRaman spectra. Therefore, the *g*_1_-value and rRaman results cannot be explained by a hydrogen bond with structural properties identical to that found in mouse R2. If a hydrogen bond to the tyrosyl radical is present in EBV R2, our spectroscopic data and DFT analysis indicate that it has characteristics more similar to that in HSV 1 R2, with a hydrogen outside of the phenoxyl plane. In contrast to mouse and HSV 1, where evidence for the involvement of a hydrogen bonded water molecule with exchangeable hydrogens has been observed, we could not clearly identify a water molecule as the hydrogen bond donor moiety for the tyrosyl radical in EBV R2. The observation of a spectroscopically different kind of tyrosyl radical in EBV R2 is a further indication for the variation of tyrosyl radicals of class Ia R2s in particular and in proteins in general. The preliminary UV/VIS spectroscopic studies carried out on EBV R2 showed that iron is most likely more weakly bound compared with *E. coli* R2, but stronger than for mouse R2. Differences can also be seen in the impact of the radical scavenger HU, which exhibits a reduced effect on the diferric center compared with mouse R2, where the reaction leads to reduction of the diferric center and dissociation of Fe^2+^. Our findings indicate that EBV R2 protein has evolved and includes a different kind of tyrosyl radical than in the seemingly simpler *E. coli* R2.

## Materials and Methods

All high-grade chemicals were purchased from Sigma or Fluka unless stated otherwise.

### Protein expression and purification

The *BaRF1* gene (accession number YP_401656; encoding full-length EBV R2) was cloned from a B95-8 derived bacterial artificial chromosome by recombinatorial cloning (Gateway™, Invitrogen) in the laboratory of Prof. Jürgen Haas (Max von Pettenkofer Institut, Gene center, Ludwig-Maximilians University, Munich). *BaRF1* was then subcloned with the LR recombination reaction into a pTH27 plasmid, with a coding sequence for an N-terminal polyhistidine tag (one letter coded amino acid sequence: MGPHHHHHHLESTSLYKKA-GSA), with the Gateway system (Invitrogen) according to the manufacturer's instructions. *E. coli* strain BL21 (DE3) (Stratagene) was transformed with the product from the LR reaction and small-scale soluble protein expression was verified as described [59], [60]. DNA sequencing (MWG Biotech, Germany) used the T7 forward and reverse promoter sequences in the plasmid as primers. Recombinant EBV R2 was produced in BL21 (DE3) *E. coli* cells in phosphate-buffered terrific broth (Formedium) supplemented with carbenicillin (Duchefa; final concentration of 50 µg/ml). Cells were grown at 37°C until an OD_600_ of 1–1.3. Protein expression was induced by addition of isopropyl-γ-D-thiogalactopyranoside (Anatrace; final concentration of 0.2 mM) and cells grown for ∼16 hrs at 18°C. The cells were harvested by centrifugation at 6500 rpm for 40 min. The cell pellet was resuspended in a buffer of 50 mM Na_2_HPO_4_/NaH_2_PO_4_, (Scharlau) pH 8.0, 500 mM NaCl (Scharlau), 10% (v/v) glycerol (Saveen Werner), 0.5 mM tris(2-carboxyethyl)phosphine (TCEP) (buffer A) containing 10 mM imidazole, 0.08 mg/ml lysozyme, 10 Units/ml DNase I recombinant (Roche), and ethylenediaminetetraacetic acid (EDTA)-free protease inhibitor tablets (Roche) (1 tablet/50 ml). Solubilized pellets were stored at 253 K until usage. Upon sonication on ice-bath, cell debris was removed by centrifugation at 65 000 rpm at 5°C and subsequent filtration (0.20 µm filter, Millipore) on ice. The soluble extract was passed over a 5 ml HisTrap FF Crude column (nickel sepharose 6 fast flow resin; GE Healthcare) at ∼10 ml/min flowrate, at 5°C , on an Äkta Express purification system (GE Healthcare). The column was washed with 10 column volumes of buffer A containing 10 mM imidazole followed by 10 column volumes of buffer A containing 70 mM imidazole. EBV R2 protein was eluted with buffer A containing 500 mM imidazole, with protease inhibitor tablets (1 tablet/50 ml). The combined elution fractions (volume *ca.* 10 ml) were gelfiltered on a Superdex S-200 HiLoad 16/60 column (GE Healthcare) on an Äkta Express system at 5°C with a buffer of 20 mM Hepes, pH 7.5, 300 mM NaCl, 10% (v/v) glycerol (buffer B), with 0.5 mM TCEP. Protein samples were concentrated with a 10 kDa molecular weight cut-off concentrator (Amicon Ultra-15, Millipore). Metalfree EBV R2 was purified analogously as described above with buffer B with 0.5 mM TCEP and 25–30 mM EDTA. The gelfiltered protein samples were left incubated 15–20 hrs in this solution, then concentrated to ∼2–3 ml, and dialysed (Slide-A-Lyzer, 7 kDa molecular weight cut-off, Pierce) twice against 5 l of buffer B at 5°C.

### Sample preparation

Protein samples of metal-free EBV R2 were concentrated up to ca. 1 mM, using the theoretical calculated absorption coefficient [61] of EBV R2 at 280 nm. Reconstitution of the dimetal-oxygen cluster and generation of the tyrosyl radical site in EBV R2 was performed by addition of Fe^2+^ ((NH_4_)_2_Fe(SO_4_)_2_·6H_2_O) in a ca. 7∶1 molar ratio to the R2 homodimer) and naturally present O_2_. All samples were incubated at 273 K for 10 minutes. The final volume of the EPR samples was ∼200 µL in 50 mM HEPES (4-(2-hydroxyethyl)-1-piperazineethanesulfonic acid) pH 7.5, 100 mM KCl, 20% (v/v) glycerol. Samples of active EBV R2 for resonance Raman spectroscopy were prepared in the same way as for EPR measurements, but the protein was first transferred to a buffer of 50 mM Tris (tris(hydroxymethyl)aminomethane)-HCl, pH 7.5, 100 mM KCl. Samples of active EBV R2 protein in deuterated buffer (50 mM Tris-HCl, 100 mM KCl, pD = 7.9) for resonance Raman spectroscopy were prepared by deuterating the metal-free protein with D_2_O (99.9%D, Cambridge Isotope Laboratories) by repeatedly diluting and re-concentrating in this buffer using Amicon Ultra-15 concentrators (Millipore) before reconstituting the diiron-oxygen cluster as explained above. The diferrous R2 samples were prepared under anaerobic conditions in air-tight vessels by several rounds of vacuum and argon exchange using the Schlenk technique. 200 µl of apo EBV R2 (150 µM) was reduced with 2 µL of 10 mM dithionite 2 µL and 5 mM methyl viologen (reductant mediator). 5 µl of the ferrous solution (42 mM) was added to fully reconstitute the dimetal site followed by incubation for 10 min at 273 K. The samples were finally transferred to anaerobic EPR tubes. Samples of EBV R2 for measurement of the mixed valence signal were prepared as described [43] both anaerobically and aerobically. 1–2 mM of dithionite was used as a reductant and 2 mM of phenazine methosulfate was used as a reductant mediator. Light absorption spectra of purified histidine-tagged EBV R2 were measured on a Hewlett-Packard 8452 diode array spectrophotometer in the wavelength range of 250–700 nm using a thermostated water bath. Experiments under anaerobic conditions were carried out in cuvettes capped with a rubber septum, which had been deaerated with argon for ≥2 hrs. Addition of deaerated solutions of EBV R2 and radical scavengers were made through a septum with gas tight syringes purged with deoxygenated water.

### EPR experiments

EPR spectra were recorded at X-band on a Bruker Elexsys 560 EPR spectrometer fitted with a Bruker ER41116DM dual-mode cavity. All EPR samples contained 20% (v/v) glycerol for vitrification during the low-temperature recordings. EPR signals were measured at different microwave powers to prevent microwave power saturation and quantified by comparing double integrals of spectra with a standard of 1 mM Cu^2+^ EDTA in a solution of 50 mM HEPES, pH 7.5, 20% (v/v) glycerol. All spectra were measured under identical non-saturating microwave power. First-derivative EPR spectra were recorded at different microwave power (P) and at various temperatures (see graphs) to determine the microwave power at half saturation (P_1/2_) for each of the temperatures. The data were fit with the function S/√P = 1/1+(P/P_1/2_))*^b^* ^/2^, where S denotes the double integrated signal intensity of the EPR signal, and *b* is a component relating to the type of relaxation. The *b* factor is equal to 1 for a completely inhomogenous relaxation and 3 for an entirely homogenous relaxation. The simulated EPR spectra (at X-band and at 285 GHz) were computed with the program SIM written by Weihe in order to extract numerical values of spin Hamiltonian parameters from experimental EPR spectra [62], [63].

### High-field EPR measurements

The low temperature 285 GHz spectra were obtained with a 95 GHz Gunn oscillator (Radiometer Physics, Germany) coupled to a frequency tripler as the frequency source and a superconducting magnet with a maximum field of 12 T at 4.2 K (Cryogenics Consultant, UK) for the main magnetic field. The detection of light transmitted through the sample was performed with a ‘hot electron’ InSb bolometer as described [32], [34].

### Resonance Raman spectroscopy

A three stage Laser system was employed as a light source for the Raman spectroscopy: a Spectra-Physics Millennia Pro 12sJS Nd∶YV0_4_ solid state laser (6.5 W at 532 nm) pumped a Sirah Matisse TR Ti∶Sa ring Laser that produced a power of 1 W at 820 nm. This was finally doubled to 410 nm using a Spectra-Physics Wavetrain frequency doubler (550–990 nm) yielding 20 mW of laser light having a line width <4 MHz. The applied power of the 410 nm laser at the sample was ∼5 mW. For the Raman measurements, a Jobyn Yvon Horiba T64000 instrument equipped with a 410 nm Kaiser Optical holographic Super-Notch filter served as a single spectrograph in order to minimize the reduction of Raman light. The narrow bandwidth and the small shifts of the Raman peaks investigated required an entrance slit width of 100 µm and resolution grating of 3600 grooves/mm. 60 scans of 60 seconds were averaged for each Raman spectrum. Data reproducibility was evaluated by triple measurements on protein samples from different preparations. The samples with a volume of ca. 50 µl were kept in EPR tubes cooled with liquid N_2_ in a quartz cold finger EPR cryostat. The fluorescence that inevitably occurred was subtracted by fitting with a polynomial function, and the frequency scale was calibrated using 4-acetamidophenol. The accuracy for the frequency of the Raman peaks was then determined within an error range of ±1.1 cm^−1^ under these settings. The reference peak values were obtained from published tables [64].

### Computational procedures

The theoretical modeling of the tyrosyl radical was carried out by density functional theory (DFT) in gas phase using a simplified system with the *p*-ethylphenoxyl radical (neutral form) using unrestricted B3LYP function (Exchange: 0.2000 Hartree-Fock, 0.0800 Slater and 0.7200 Becke, and correlation 0.8100 LYP and 0.1900 VWN1RPA) with the Euler-Maclaurin-Lebedev (70,302) grid and basis set 6-31G(d,p) as implemented in the computational package Spartan 08/10. The molecular structures in the presence and absence of an H-bonded water molecule were fully optimized (root mean square gradient below 10^−7^) with and without C6-C1-Cα-Cβ torsional angle (constraint θ) followed by frequency calculation in order to derive vibrational frequencies and Raman scattering activities (unscaled). More accurate spin-densities used for the estimation of the hyperfine A_H_ tensor components in EBV R2 have been derived by using the entire tyrosyl radical without and with a water molecule located at 2.60 Å from the tyrosyl oxygen by UB3LYP/6-311++G(d,p) complemented by Raman frequency calculations at the same theory level. Details are provided in the Information S1 and methods section.

### UV/VIS light spectrophotometric assays

Light absorption spectra of purified histidine-tagged EBV R2 were measured on a Hewlett-Packard 8452 diode array spectrophotometer in the wavelength range of 250–700 nm using a thermostated water bath. Experiments under anaerobic conditions were carried out in cuvettes capped with a rubber septum, which had been deaerated with argon for ≥2 hrs. Addition of deaerated solutions of EBV R2 and radical scavengers were made through a septum with gas tight syringes purged with deoxygenated water.

## Supporting Information

Information S1 **Theoretical DFT Analyses.**
(DOC)
