# Supplementary material for: HF-EPR, Raman, UV/VIS Light Spectroscopic, and DFT Studies of the Ribonucleotide Reductase R2 Tyrosyl Radical from Epstein-Barr Virus
Source: PLoS One. 2011 Sep 27;6(9):e25022. doi: 10.1371/journal.pone.0025022 (PMC3181257; doi:10.1371/journal.pone.0025022)
Supplement: Information S1 — Theoretical DFT Analyses. (DOC) [file pone.0025022.s001.doc]

**Supporting material**

**HF-EPR, Raman, UV/VIS Light Spectroscopic and DFT Studies of the Ribonucleotide Reductase R2 Tyrosyl Radical from Epstein-Barr Virus**

Ane B. Tomter, Giorgio Zoppellaro, Florian Schmitzberger, Niels H. Andersen, Anne-Laure Barra, Henrik Engman, Pär Nordlund and K. Kristoffer Andersson*

**PART I: Theoretical DFT Analyses**

The computational works were carried on using Spartan 08/10, ver. 1.2.0 (Wavefunction and Q-Chem Inc.) on HP Compaq DC5800.

**From Figure 2A.** Method: geometry opt under constraint angle (θ = 30 deg): UB3LYP/6-311++G**, Euler-Maclaurin-Lebedev (EML) grid, containing 70 radial shells with 302 angular points per shell, neutral form, doublet. In blue the value of the spin-density for the phenoxyl oxygen and in red the C(4) atom.


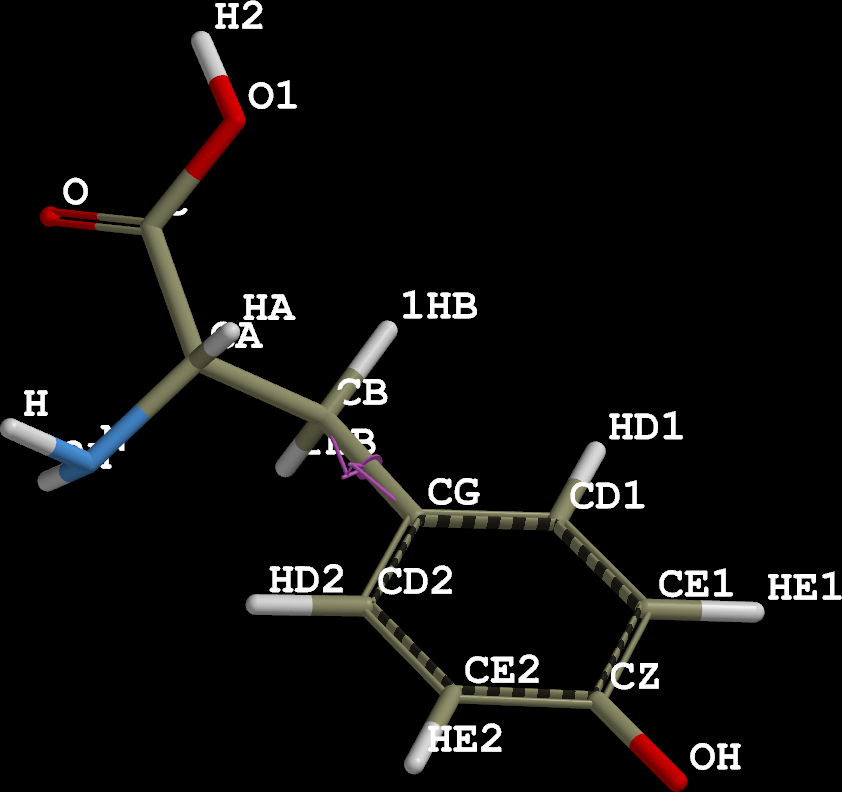


**Natural Atomic Populations and Charges**

**Alpha spin Beta spin Spin**

**Atom Occupancy Occupancy Density Charge**

**------ ----------- ----------- ----------- ------------**

**N 3.920140 3.918615 0.001525 -0.838755**

**CA 3.059298 3.053848 0.005450 -0.113147**

**C 2.601154 2.600374 0.000780 0.798473**

**O 4.300272 4.299710 0.000562 -0.599982**

**2H 0.317387 0.317438 -0.000051 0.365175**

**H 0.318575 0.318584 -0.000009 0.362842**

**HA 0.390519 0.390878 -0.000358 0.218603**

**CB 3.196828 3.212486 -0.015657 -0.409314**

**CG 3.148221 2.802594 0.345627 0.049186**

**1HB 0.390727 0.385640 0.005087 0.223633**

**2HB 0.399405 0.377024 0.022381 0.223570**

**CZ 2.791886 2.850863 -0.058977 0.357251**

**CD1 3.054096 3.167361 -0.113265 -0.221457**

**CD2 3.058893 3.174538 -0.115645 -0.233431**

**CE2 3.217760 2.952247 0.265514 -0.170007**

**CE1 3.217322 2.956868 0.260455 -0.174190**

**HD1 0.398647 0.395586 0.003061 0.205767**

**HD2 0.382495 0.379429 0.003065 0.238076**

**HE2 0.388641 0.396678 -0.008038 0.214681**

**HE1 0.388073 0.395970 -0.007897 0.215956**

**OH 4.455276 4.049195 0.406081 -0.504472**

**O1 4.347108 4.346791 0.000316 -0.693899**

**H2 0.257278 0.257283 -0.000006 0.485439**

**Total Charge = 0.00**

**From Figure 2B.** Method: geometry opt (θ = 30 deg): UB3LYP/6-311++G**, Euler-Maclaurin-Lebedev (EML) grid, containing 70 radial shells with 302 angular points per shell, neutral form, doublet. In blue the value of the spin-density for the phenoxyl oxygen and in red the C(4) atom.


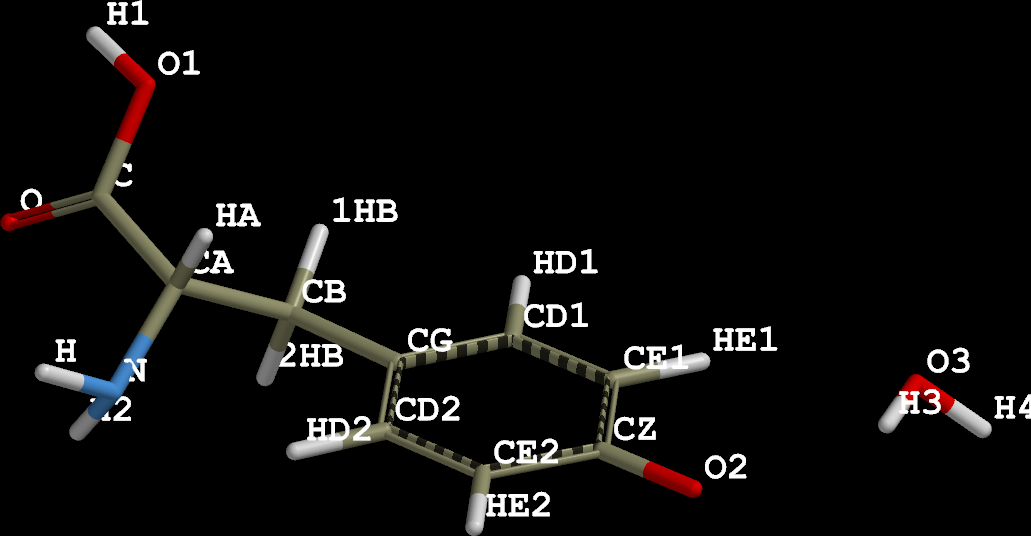


**Natural Atomic Populations and Charges**

**Alpha spin Beta spin Spin**

**Atom Occupancy Occupancy Density Charge**

**------ ----------- ----------- ----------- ------------**

**N 3.920236 3.918636 0.001600 -0.838872**

**CA 3.059299 3.053902 0.005397 -0.113202**

**C 2.601127 2.600328 0.000798 0.798545**

**O 4.300304 4.299734 0.000570 -0.600037**

**H 0.318560 0.318567 -0.000007 0.362873**

**HA 0.390563 0.390923 -0.000359 0.218514**

**CB 3.197319 3.212842 -0.015523 -0.410160**

**CG 3.146415 2.800558 0.345856 0.053027**

**1HB 0.390138 0.384985 0.005153 0.224877**

**2HB 0.399373 0.376922 0.022452 0.223705**

**CZ 2.796921 2.840729 -0.043808 0.362350**

**CD1 3.053613 3.167430 -0.113817 -0.221043**

**CD2 3.061077 3.171142 -0.110065 -0.232220**

**CE2 3.216064 2.958014 0.258050 -0.174078**

**CE1 3.216953 2.958742 0.258211 -0.175695**

**HD1 0.398169 0.395124 0.003045 0.206706**

**HD2 0.382315 0.379422 0.002893 0.238263**

**HE2 0.389023 0.396850 -0.007827 0.214126**

**HE1 0.377050 0.384645 -0.007594 0.238305**

**O1 4.347026 4.346715 0.000311 -0.693741**

**H1 0.257231 0.257237 -0.000007 0.485532**

**H2 0.317499 0.317554 -0.000054 0.364947**

**H3 0.260696 0.260854 -0.000158 0.478451**

**O3 4.468523 4.468710 -0.000187 -0.937233**

**H4 0.270179 0.270220 -0.000042 0.459601**

**O2 4.464327 4.069216 0.395111 -0.533543**

**Total Charge = 0.00**

**From Figure 2.** Calculated Raman spectra of a tyrosyl radical (neutral form, gas phase, 8 cm^-1^ Lorentzian line band-width) without (A) and with (B) a water molecule close to the phenoxyl oxygen (on the phenoxyl plane at distance of 2.60 Å). Method: UB3LYP/6-311++G**, Euler-Maclaurin-Lebedev (EML) grid, containing 70 radial shells with 302 angular points per shell, neutral form, doublet. The ν_7a_ mode (Wilson notation, C-O stretching) is evidenced with a red arrow in each panel.

**
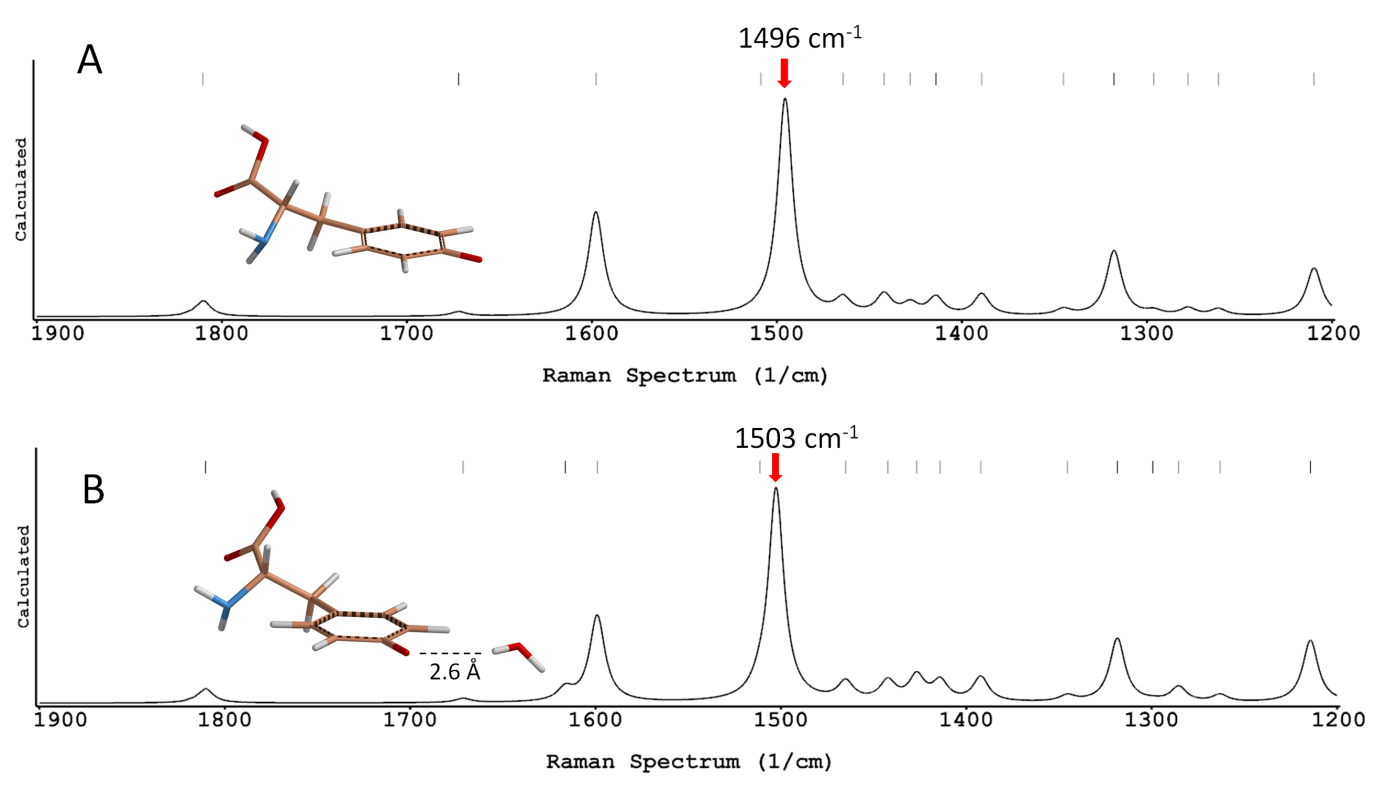
**

**From Figure 2A: Complete list of vibrational frequencies (UB3LYP/6-311++G**).** Note due to constrain on θ (30 deg) one imaginary frequency has been found. The mode 43 corresponds to the C-O stretching vibration.

**************************************************************************

**** ****

**** VIBRATIONAL ANALYSIS ****

**** -------------------- ****

**** ****

**** VIBRATIONAL FREQUENCIES (CM**-1) AND NORMAL MODES ****

**** FORCE CONSTANTS (mDYN/ANGSTROM) AND REDUCED MASSES (AMU) ****

**** INFRARED INTENSITIES (KM/MOL) ****

**** RAMAN SCATTERING ACTIVITIES (A**4/AMU) AND DEPOLARIZATION RATIOS ****

**** ****

**************************************************************************

**Mode: 1 2 3**

**Frequency: -17.57 38.00 46.55**

**Force Cnst: 0.0006 0.0082 0.0084**

**Red. Mass: 3.5461 9.5807 6.5423**

**IR Active: YES YES YES**

**IR Intens: 0.140 3.199 1.730**

**Raman Active: YES YES YES**

**Raman Intens: 3.257 0.742 3.444**

**Depolar: 0.724 0.673 0.749**

**Mode: 4 5 6**

**Frequency: 100.98 145.40 193.29**

**Force Cnst: 0.0367 0.0533 0.1203**

**Red. Mass: 6.1167 4.2806 5.4643**

**IR Active: YES YES YES**

**IR Intens: 1.383 1.836 0.220**

**Raman Active: YES YES YES**

**Raman Intens: 0.861 0.253 1.344**

**Depolar: 0.686 0.654 0.278**

**Mode: 7 8 9**

**Frequency: 243.93 269.69 296.36**

**Force Cnst: 0.0447 0.1945 0.1630**

**Red. Mass: 1.2746 4.5394 3.1505**

**IR Active: YES YES YES**

**IR Intens: 23.718 10.545 11.801**

**Raman Active: YES YES YES**

**Raman Intens: 0.850 1.366 0.423**

**Depolar: 0.532 0.317 0.255**

**Mode: 10 11 12**

**Frequency: 304.62 380.63 389.65**

**Force Cnst: 0.2079 0.2677 0.2755**

**Red. Mass: 3.8017 3.1356 3.0799**

**IR Active: YES YES YES**

**IR Intens: 3.867 0.298 7.017**

**Raman Active: YES YES YES**

**Raman Intens: 1.934 0.640 3.239**

**Depolar: 0.679 0.273 0.155**

**Mode: 13 14 15**

**Frequency: 452.91 486.07 488.14**

**Force Cnst: 0.6355 0.6527 0.3992**

**Red. Mass: 5.2584 4.6887 2.8437**

**IR Active: YES YES YES**

**IR Intens: 4.100 13.979 6.109**

**Raman Active: YES YES YES**

**Raman Intens: 1.981 6.655 0.518**

**Depolar: 0.222 0.338 0.362**

**Mode: 16 17 18**

**Frequency: 542.37 595.66 623.55**

**Force Cnst: 0.5470 0.2858 1.1174**

**Red. Mass: 3.1559 1.3669 4.8777**

**IR Active: YES YES YES**

**IR Intens: 16.769 98.586 16.883**

**Raman Active: YES YES YES**

**Raman Intens: 0.918 1.036 6.547**

**Depolar: 0.513 0.682 0.740**

**Mode: 19 20 21**

**Frequency: 656.61 703.54 731.96**

**Force Cnst: 0.9671 0.8674 1.2223**

**Red. Mass: 3.8073 2.9744 3.8723**

**IR Active: YES YES YES**

**IR Intens: 12.657 6.533 13.563**

**Raman Active: YES YES YES**

**Raman Intens: 5.082 1.327 0.265**

**Depolar: 0.435 0.122 0.573**

**Mode: 22 23 24**

**Frequency: 792.02 800.36 816.60**

**Force Cnst: 1.2750 0.4929 2.1234**

**Red. Mass: 3.4497 1.3060 5.4045**

**IR Active: YES YES YES**

**IR Intens: 28.177 1.202 5.972**

**Raman Active: YES YES YES**

**Raman Intens: 5.914 1.463 13.784**

**Depolar: 0.061 0.210 0.080**

**Mode: 25 26 27**

**Frequency: 849.34 855.47 893.69**

**Force Cnst: 0.8450 1.0385 0.9858**

**Red. Mass: 1.9882 2.4086 2.0948**

**IR Active: YES YES YES**

**IR Intens: 24.704 82.505 30.842**

**Raman Active: YES YES YES**

**Raman Intens: 0.505 4.260 3.950**

**Depolar: 0.720 0.095 0.508**

**Mode: 28 29 30**

**Frequency: 948.14 976.57 990.35**

**Force Cnst: 1.2567 0.8082 1.1389**

**Red. Mass: 2.3727 1.4383 1.9708**

**IR Active: YES YES YES**

**IR Intens: 64.807 4.974 8.817**

**Raman Active: YES YES YES**

**Raman Intens: 22.334 1.078 1.970**

**Depolar: 0.152 0.600 0.526**

**Mode: 31 32 33**

**Frequency: 992.06 1013.95 1116.13**

**Force Cnst: 1.5969 0.7985 1.1126**

**Red. Mass: 2.7540 1.3183 1.5158**

**IR Active: YES YES YES**

**IR Intens: 1.679 2.498 7.524**

**Raman Active: YES YES YES**

**Raman Intens: 24.610 0.128 2.027**

**Depolar: 0.334 0.750 0.293**

**Mode: 34 35 36**

**Frequency: 1124.16 1154.83 1168.06**

**Force Cnst: 1.9423 1.4863 1.1124**

**Red. Mass: 2.6086 1.8916 1.3839**

**IR Active: YES YES YES**

**IR Intens: 183.667 73.795 39.043**

**Raman Active: YES YES YES**

**Raman Intens: 2.612 5.228 4.398**

**Depolar: 0.674 0.460 0.384**

**Mode: 37 38 39**

**Frequency: 1170.55 1209.66 1261.34**

**Force Cnst: 1.0138 2.0880 1.6656**

**Red. Mass: 1.2558 2.4219 1.7769**

**IR Active: YES YES YES**

**IR Intens: 1.072 7.155 8.320**

**Raman Active: YES YES YES**

**Raman Intens: 2.689 32.316 4.417**

**Depolar: 0.517 0.156 0.120**

**Mode: 40 41 42**

**Frequency: 1277.75 1296.31 1317.77**

**Force Cnst: 2.0638 1.8057 1.4590**

**Red. Mass: 2.1455 1.8238 1.4260**

**IR Active: YES YES YES**

**IR Intens: 4.958 3.551 4.114**

**Raman Active: YES YES YES**

**Raman Intens: 4.966 2.805 43.696**

**Depolar: 0.724 0.497 0.429**

**Mode: 43 44 45**

**Frequency: 1345.11 1389.31 1414.04**

**Force Cnst: 1.9566 1.7914 1.6640**

**Red. Mass: 1.8354 1.5753 1.4124**

**IR Active: YES YES YES**

**IR Intens: 29.808 2.866 6.553**

**Raman Active: YES YES YES**

**Raman Intens: 4.130 14.240 11.824**

**Depolar: 0.602 0.354 0.433**

**Mode: 46 47 48**

**Frequency: 1427.88 1442.07 1464.21**

**Force Cnst: 3.2522 2.9911 1.4350**

**Red. Mass: 2.7073 2.4412 1.1360**

**IR Active: YES YES YES**

**IR Intens: 0.539 4.519 8.871**

**Raman Active: YES YES YES**

**Raman Intens: 7.120 13.450 10.116**

**Depolar: 0.327 0.673 0.594**

**Mode: 49 50 51**

**Frequency: 1495.50 1508.68 1597.82**

**Force Cnst: 4.7290 5.0239 7.3953**

**Red. Mass: 3.5888 3.7462 4.9165**

**IR Active: YES YES YES**

**IR Intens: 48.922 4.108 114.331**

**Raman Active: YES YES YES**

**Raman Intens: 145.829 0.358 70.185**

**Depolar: 0.242 0.606 0.749**

**Mode: 52 53 54**

**Frequency: 1672.02 1810.26 2991.33**

**Force Cnst: 1.7967 19.2564 5.6445**

**Red. Mass: 1.0908 9.9733 1.0706**

**IR Active: YES YES YES**

**IR Intens: 32.831 350.294 7.140**

**Raman Active: YES YES YES**

**Raman Intens: 2.986 10.804 214.025**

**Depolar: 0.694 0.220 0.138**

**Mode: 55 56 57**

**Frequency: 3053.03 3075.52 3153.24**

**Force Cnst: 5.9521 6.0734 6.3783**

**Red. Mass: 1.0838 1.0898 1.0888**

**IR Active: YES YES YES**

**IR Intens: 8.860 7.570 10.909**

**Raman Active: YES YES YES**

**Raman Intens: 35.961 48.839 71.735**

**Depolar: 0.299 0.253 0.415**

**Mode: 58 59 60**

**Frequency: 3183.14 3191.22 3197.95**

**Force Cnst: 6.4977 6.5613 6.5970**

**Red. Mass: 1.0884 1.0935 1.0948**

**IR Active: YES YES YES**

**IR Intens: 1.831 3.937 6.388**

**Raman Active: YES YES YES**

**Raman Intens: 72.826 115.238 151.652**

**Depolar: 0.599 0.228 0.119**

**Mode: 61 62 63**

**Frequency: 3497.42 3569.74 3745.67**

**Force Cnst: 7.5661 8.2026 8.8000**

**Red. Mass: 1.0498 1.0925 1.0646**

**IR Active: YES YES YES**

**IR Intens: 4.551 7.258 77.604**

**Raman Active: YES YES YES**

**Raman Intens: 137.128 60.012 205.331**

**Depolar: 0.091 0.667 0.263**

**From Figure 2B: Complete list of vibrational frequencies (UB3LYP/6-311++G**).** Note due to constrain on θ (30 deg) and on the distance (0.26 nm) with the water molecule, one imaginary frequency has been found. The mode 55 corresponds to the C-O stretching vibration.

**************************************************************************

**** ****

**** VIBRATIONAL ANALYSIS ****

**** -------------------- ****

**** ****

**** VIBRATIONAL FREQUENCIES (CM**-1) AND NORMAL MODES ****

**** FORCE CONSTANTS (mDYN/ANGSTROM) AND REDUCED MASSES (AMU) ****

**** INFRARED INTENSITIES (KM/MOL) ****

**** RAMAN SCATTERING ACTIVITIES (A**4/AMU) AND DEPOLARIZATION RATIOS ****

**** ****

**************************************************************************

**Mode: 1 2 3**

**Frequency: -59.39 7.77 34.14**

**Force Cnst: 0.0082 0.0001 0.0040**

**Red. Mass: 3.9548 4.0439 5.7608**

**IR Active: YES YES YES**

**IR Intens: 16.100 0.210 3.236**

**Raman Active: YES YES YES**

**Raman Intens: 1.915 1.318 1.051**

**Depolar: 0.685 0.712 0.686**

**Mode: 4 5 6**

**Frequency: 38.81 50.19 63.36**

**Force Cnst: 0.0052 0.0082 0.0169**

**Red. Mass: 5.8789 5.5036 7.1458**

**IR Active: YES YES YES**

**IR Intens: 3.012 4.048 12.662**

**Raman Active: YES YES YES**

**Raman Intens: 1.750 3.192 0.151**

**Depolar: 0.741 0.742 0.713**

**Mode: 7 8 9**

**Frequency: 101.67 115.69 144.16**

**Force Cnst: 0.0319 0.0091 0.0472**

**Red. Mass: 5.2334 1.1510 3.8508**

**IR Active: YES YES YES**

**IR Intens: 1.763 107.738 4.163**

**Raman Active: YES YES YES**

**Raman Intens: 0.811 0.949 0.379**

**Depolar: 0.707 0.530 0.700**

**Mode: 10 11 12**

**Frequency: 193.46 227.21 241.82**

**Force Cnst: 0.1186 0.0370 0.0422**

**Red. Mass: 5.3772 1.2172 1.2259**

**IR Active: YES YES YES**

**IR Intens: 1.427 116.063 37.795**

**Raman Active: YES YES YES**

**Raman Intens: 1.257 1.706 0.881**

**Depolar: 0.236 0.691 0.510**

**Mode: 13 14 15**

**Frequency: 266.73 295.90 307.93**

**Force Cnst: 0.1642 0.1661 0.2030**

**Red. Mass: 3.9174 3.2190 3.6332**

**IR Active: YES YES YES**

**IR Intens: 18.458 11.962 3.934**

**Raman Active: YES YES YES**

**Raman Intens: 1.295 0.535 1.640**

**Depolar: 0.320 0.218 0.667**

**Mode: 16 17 18**

**Frequency: 351.67 384.09 390.91**

**Force Cnst: 0.0847 0.2720 0.2692**

**Red. Mass: 1.1628 3.1292 2.9902**

**IR Active: YES YES YES**

**IR Intens: 228.282 1.469 15.017**

**Raman Active: YES YES YES**

**Raman Intens: 2.295 0.763 3.767**

**Depolar: 0.669 0.298 0.163**

**Mode: 19 20 21**

**Frequency: 456.73 487.85 491.11**

**Force Cnst: 0.6495 0.7118 0.3917**

**Red. Mass: 5.2847 5.0760 2.7563**

**IR Active: YES YES YES**

**IR Intens: 1.567 20.850 0.520**

**Raman Active: YES YES YES**

**Raman Intens: 2.209 4.628 2.216**

**Depolar: 0.184 0.426 0.233**

**Mode: 22 23 24**

**Frequency: 546.40 593.40 623.14**

**Force Cnst: 0.5702 0.2793 1.1629**

**Red. Mass: 3.2415 1.3464 5.0829**

**IR Active: YES YES YES**

**IR Intens: 16.860 102.634 15.229**

**Raman Active: YES YES YES**

**Raman Intens: 0.840 1.013 7.432**

**Depolar: 0.555 0.716 0.714**

**Mode: 25 26 27**

**Frequency: 656.42 704.97 733.24**

**Force Cnst: 0.9612 0.9120 1.2229**

**Red. Mass: 3.7860 3.1146 3.8607**

**IR Active: YES YES YES**

**IR Intens: 12.104 6.885 12.143**

**Raman Active: YES YES YES**

**Raman Intens: 5.112 1.801 0.238**

**Depolar: 0.411 0.118 0.666**

**Mode: 28 29 30**

**Frequency: 792.55 812.94 818.32**

**Force Cnst: 1.3937 0.4926 2.0774**

**Red. Mass: 3.7658 1.2652 5.2653**

**IR Active: YES YES YES**

**IR Intens: 30.343 0.875 5.231**

**Raman Active: YES YES YES**

**Raman Intens: 5.538 1.039 14.742**

**Depolar: 0.091 0.440 0.074**

**Mode: 31 32 33**

**Frequency: 852.64 855.06 897.50**

**Force Cnst: 0.8398 1.0273 0.9821**

**Red. Mass: 1.9607 2.3848 2.0695**

**IR Active: YES YES YES**

**IR Intens: 24.480 81.992 35.337**

**Raman Active: YES YES YES**

**Raman Intens: 0.870 3.889 4.712**

**Depolar: 0.394 0.091 0.455**

**Mode: 34 35 36**

**Frequency: 948.95 985.62 994.99**

**Force Cnst: 1.2739 1.1276 1.7533**

**Red. Mass: 2.4011 1.9701 3.0059**

**IR Active: YES YES YES**

**IR Intens: 64.961 13.299 1.371**

**Raman Active: YES YES YES**

**Raman Intens: 23.758 2.136 25.378**

**Depolar: 0.157 0.592 0.336**

**Mode: 37 38 39**

**Frequency: 1000.45 1014.99 1122.15**

**Force Cnst: 0.8130 0.7974 1.8658**

**Red. Mass: 1.3787 1.3137 2.5149**

**IR Active: YES YES YES**

**IR Intens: 0.691 2.412 112.127**

**Raman Active: YES YES YES**

**Raman Intens: 0.406 0.127 2.244**

**Depolar: 0.741 0.742 0.250**

**Mode: 40 41 42**

**Frequency: 1127.71 1155.33 1168.22**

**Force Cnst: 1.1936 1.4756 1.1313**

**Red. Mass: 1.5930 1.8764 1.4069**

**IR Active: YES YES YES**

**IR Intens: 83.255 70.411 39.793**

**Raman Active: YES YES YES**

**Raman Intens: 3.432 5.424 3.946**

**Depolar: 0.691 0.464 0.397**

**Mode: 43 44 45**

**Frequency: 1179.91 1214.34 1263.07**

**Force Cnst: 1.0707 1.9754 1.5752**

**Red. Mass: 1.3053 2.2736 1.6758**

**IR Active: YES YES YES**

**IR Intens: 0.761 6.410 6.808**

**Raman Active: YES YES YES**

**Raman Intens: 2.543 44.493 5.150**

**Depolar: 0.556 0.165 0.130**

**Mode: 46 47 48**

**Frequency: 1285.42 1299.42 1318.51**

**Force Cnst: 2.4877 1.6744 1.4606**

**Red. Mass: 2.5554 1.6831 1.4259**

**IR Active: YES YES YES**

**IR Intens: 7.886 2.404 4.442**

**Raman Active: YES YES YES**

**Raman Intens: 10.691 0.648 45.537**

**Depolar: 0.545 0.619 0.429**

**Mode: 49 50 51**

**Frequency: 1345.42 1392.14 1414.07**

**Force Cnst: 1.9408 1.8093 1.6707**

**Red. Mass: 1.8198 1.5845 1.4181**

**IR Active: YES YES YES**

**IR Intens: 29.685 2.576 7.432**

**Raman Active: YES YES YES**

**Raman Intens: 4.373 17.647 14.077**

**Depolar: 0.634 0.321 0.414**

**Mode: 52 53 54**

**Frequency: 1426.76 1442.35 1465.10**

**Force Cnst: 3.3555 2.9700 1.4310**

**Red. Mass: 2.7977 2.4231 1.1315**

**IR Active: YES YES YES**

**IR Intens: 0.078 4.955 10.383**

**Raman Active: YES YES YES**

**Raman Intens: 17.788 14.103 13.276**

**Depolar: 0.294 0.683 0.487**

**Mode: 55 56 57**

**Frequency: 1502.59 1511.42 1599.08**

**Force Cnst: 4.2307 4.8217 7.3407**

**Red. Mass: 3.1804 3.5824 4.8724**

**IR Active: YES YES YES**

**IR Intens: 78.622 5.976 116.746**

**Raman Active: YES YES YES**

**Raman Intens: 151.872 6.196 61.977**

**Depolar: 0.263 0.317 0.748**

**Mode: 58 59 60**

**Frequency: 1616.34 1671.41 1810.47**

**Force Cnst: 1.6648 1.7958 19.3013**

**Red. Mass: 1.0815 1.0911 9.9944**

**IR Active: YES YES YES**

**IR Intens: 82.485 32.844 352.662**

**Raman Active: YES YES YES**

**Raman Intens: 8.667 2.997 10.458**

**Depolar: 0.705 0.699 0.223**

**Mode: 61 62 63**

**Frequency: 2990.52 3052.05 3076.18**

**Force Cnst: 5.6423 5.9481 6.0744**

**Red. Mass: 1.0708 1.0838 1.0895**

**IR Active: YES YES YES**

**IR Intens: 6.895 8.987 6.933**

**Raman Active: YES YES YES**

**Raman Intens: 218.159 36.336 49.622**

**Depolar: 0.140 0.285 0.246**

**Mode: 64 65 66**

**Frequency: 3154.76 3182.93 3185.79**

**Force Cnst: 6.3855 6.4973 6.5471**

**Red. Mass: 1.0890 1.0885 1.0949**

**IR Active: YES YES YES**

**IR Intens: 12.281 2.408 10.058**

**Raman Active: YES YES YES**

**Raman Intens: 70.923 76.113 153.799**

**Depolar: 0.449 0.527 0.229**

**Mode: 67 68 69**

**Frequency: 3198.18 3497.47 3569.88**

**Force Cnst: 6.5977 7.5664 8.2030**

**Red. Mass: 1.0948 1.0499 1.0925**

**IR Active: YES YES YES**

**IR Intens: 5.972 4.556 7.097**

**Raman Active: YES YES YES**

**Raman Intens: 140.397 140.212 61.857**

**Depolar: 0.120 0.093 0.658**

**Mode: 70 71 72**

**Frequency: 3745.68 3785.96 3902.66**

**Force Cnst: 8.8002 8.8515 9.6910**

**Red. Mass: 1.0646 1.0481 1.0799**

**IR Active: YES YES YES**

**IR Intens: 78.179 45.909 103.769**

**Raman Active: YES YES YES**

**Raman Intens: 207.656 181.040 46.293**

**Depolar: 0.263 0.166 0.485**

**Spin and charge distribution calculated for the ethylphenoxyl radical**

**From Figure [5A].** Method: geometry opt+freq calculation: UB3LYP/6-31G(d,p), Euler-Maclaurin-Lebedev (EML) grid, containing 70 radial shells with 302 angular points per shell, neutral form, doublet. In blue the value of the spin-density for the phenoxyl oxygen and in red the C(4) atom.


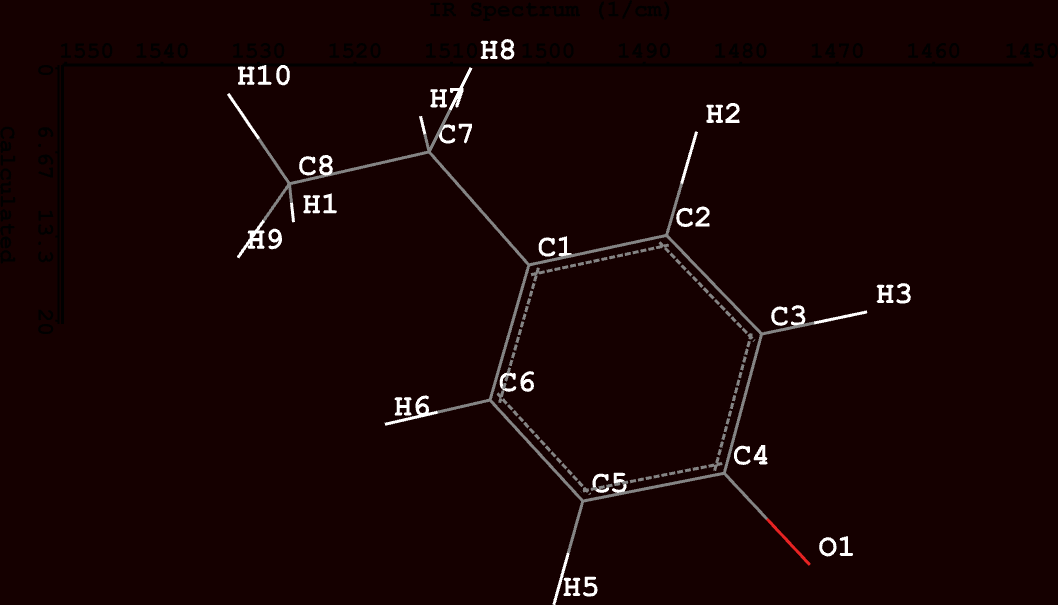


Natural Atomic Populations and Charges (similar to what is used by Himo, F., A. Graslund, and L. A. Eriksson. Density functional calculations on model tyrosyl radicals. *Biophys. J.* **1997**,*72*,1556-1567)

Alpha spin Beta spin Spin

Atom Occupancy Occupancy Density Charge

------ ----------- ----------- ----------- ------------

C1 3.152481 2.809504 0.342976 0.038015

**C4 2.778838 2.851527 -0.072689 0.369635**

C2 3.067670 3.184944 -0.117274 -0.252613

C6 3.070364 3.189321 -0.118957 -0.259685

C5 3.244809 2.972336 0.272473 -0.217145

C3 3.239945 2.981363 0.258582 -0.221309

H2 0.380438 0.377236 0.003202 0.242326

H6 0.379716 0.376498 0.003219 0.243786

H5 0.371122 0.379318 -0.008196 0.249560

H3 0.371015 0.378840 -0.007825 0.250145

C7 3.237042 3.251357 -0.014314 -0.488399

H7 0.380735 0.360083 0.020652 0.259182

H8 0.378303 0.372938 0.005366 0.248759

C8 3.347179 3.342464 0.004715 -0.689644

H1 0.380825 0.380616 0.000209 0.238558

H9 0.384002 0.384165 -0.000163 0.231833

H10 0.377519 0.377375 0.000144 0.245106

**O1 4.457996 4.030113 0.427883 -0.488109**

Ground-State Mulliken Net Atomic Charges (similar to what is used by Svistunenko, D. A., and G. A. Jones. Tyrosyl radicals in proteins: a comparison of empirical and density functional calculated EPR parameters. *PhysChemChemPhys* **2009**, *11*, 6600-6613)

Atom Charge (a.u.) Spin (a.u.)

--------------------------------------------------------

1 C 0.126959 0.397531

**2 C 0.381652 -0.105000**

3 C -0.116965 -0.159778

4 C -0.108565 -0.163279

5 C -0.109203 0.314002

6 C -0.103637 0.298285

7 H 0.090655 0.005313

8 H 0.093663 0.005413

9 H 0.108972 -0.013987

10 H 0.105966 -0.013329

11 C -0.244932 -0.028531

12 H 0.147670 0.023218

13 H 0.105452 0.006450

14 C -0.327644 0.005128

15 H 0.101737 0.001024

16 H 0.122911 -0.000292

17 H 0.112073 0.000589

**18 O -0.486764 0.427245**

--------------------------------------------------------

Sum of atomic charges = 0.000000

Sum of spin charges = 1.000000

**From Figure [5b].** Method: geometry opt+freq calculation: UB3LYP/6-31G(d,p), Euler-Maclaurin-Lebedev (EML) grid, containing 70 radial shells with 302 angular points per shell, neutral form, doublet. In blue the value of the spin-density for the phenoxyl oxygen and in red the C(4) atom.


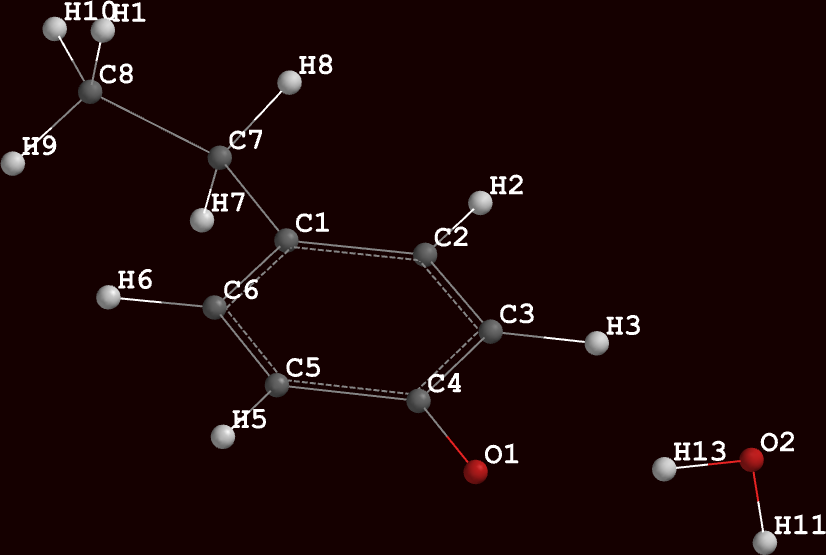


Natural Atomic Populations and Charges

Alpha spin Beta spin Spin

Atom Occupancy Occupancy Density Charge

------ ----------- ----------- ----------- ------------

C1 3.148845 2.803483 0.345361 0.047672

**C4 2.788274 2.829409 -0.041135 0.382317**

C2 3.068262 3.182184 -0.113922 -0.250446

C6 3.073432 3.183678 -0.110247 -0.257110

C5 3.238094 2.978246 0.259848 -0.216339

C3 3.236160 2.985796 0.250365 -0.221956

H2 0.378908 0.375854 0.003054 0.245239

H6 0.378633 0.375686 0.002947 0.245682

H5 0.370254 0.378092 -0.007837 0.251654

H3 0.361360 0.368512 -0.007153 0.270128

C7 3.238201 3.252067 -0.013866 -0.490269

H7 0.379968 0.358887 0.021081 0.261145

H8 0.377219 0.371733 0.005485 0.251048

C8 3.347512 3.342729 0.004783 -0.690241

H1 0.380417 0.380200 0.000218 0.239383

H9 0.383962 0.384112 -0.000150 0.231927

H10 0.376852 0.376685 0.000167 0.246463

**O1 4.463704 4.060605 0.403100 -0.524309**

O2 4.491952 4.492968 -0.001016 -0.984920

H11 0.265942 0.265962 -0.000020 0.468096

H13 0.252051 0.253113 -0.001062 0.494837

Ground-State Mulliken Net Atomic Charges

Atom Charge (a.u.) Spin (a.u.)

--------------------------------------------------------

1 C 0.135920 0.399909

**2 C 0.380988 -0.071465**

3 C -0.122646 -0.158355

4 C -0.111708 -0.154066

5 C -0.098279 0.299901

6 C -0.116895 0.289192

7 H 0.098104 0.005180

8 H 0.093400 0.004985

9 H 0.111314 -0.013335

10 H 0.137762 -0.011991

11 C -0.249387 -0.028829

12 H 0.113801 0.023770

13 H 0.125492 0.006583

14 C -0.326307 0.005252

15 H 0.138162 0.000848

16 H 0.104152 -0.000284

17 H 0.110974 0.000660

**18 O -0.505289 0.405642**

19 O -0.631662 -0.000721

20 H 0.284458 -0.000047

21 H 0.327648 -0.002829

--------------------------------------------------------

Sum of atomic charges = 0.000000

Sum of spin charges = 1.000000

**From Figure [5C].** Method: geometry opt+freq calculation: UB3LYP/6-31G(d,p), Euler-Maclaurin-Lebedev (EML) grid, containing 70 radial shells with 302 angular points per shell,neutral form, doublet. In blue the value of the spin-density for the phenoxyl oxygen and in red the C(4) atom.


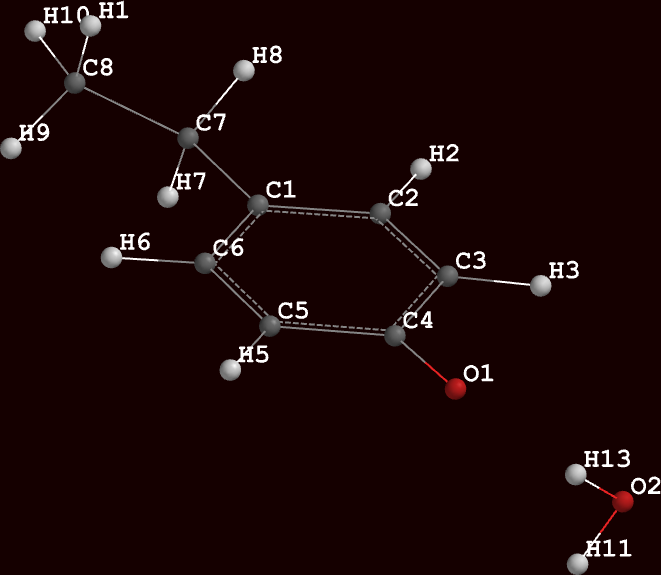


Natural Atomic Populations and Charges

Alpha spin Beta spin Spin

Atom Occupancy Occupancy Density Charge

------ ----------- ----------- ----------- ------------

C1 3.148543 2.803633 0.344910 0.047824

**C4 2.789093 2.829936 -0.040843 0.380971**

C2 3.069010 3.182842 -0.113832 -0.251853

C6 3.073860 3.183283 -0.109423 -0.257143

C5 3.238383 2.979575 0.258808 -0.217959

C3 3.232850 2.982767 0.250083 -0.215617

H2 0.378651 0.375581 0.003070 0.245769

H6 0.378545 0.375632 0.002912 0.245823

H5 0.370311 0.378108 -0.007798 0.251581

H3 0.362556 0.369976 -0.007420 0.267468

C7 3.238218 3.252075 -0.013857 -0.490293

H7 0.379753 0.358747 0.021006 0.261499

H8 0.377212 0.371764 0.005448 0.251024

C8 3.347638 3.342791 0.004848 -0.690429

H1 0.380449 0.380245 0.000204 0.239306

H9 0.383900 0.384058 -0.000158 0.232042

H10 0.376739 0.376560 0.000179 0.246701

**O1 4.462545 4.062997 0.399548 -0.525542**

O2 4.491383 4.487848 0.003535 -0.979231

H11 0.267162 0.267323 -0.000161 0.465514

H13 0.253200 0.254257 -0.001057 0.492542

Ground-State Mulliken Net Atomic Charges

Atom Charge (a.u.) Spin (a.u.)

--------------------------------------------------------

1 C 0.134915 0.398929

**2 C 0.373093 -0.072387**

3 C -0.123092 -0.159159

4 C -0.112264 -0.153154

5 C -0.097780 0.299301

6 C -0.097336 0.289773

7 H 0.100175 0.005220

8 H 0.090523 0.004958

9 H 0.109848 -0.013316

10 H 0.141928 -0.012374

11 C -0.248625 -0.028833

12 H 0.114467 0.023554

13 H 0.126182 0.006511

14 C -0.326339 0.005287

15 H 0.137101 0.000845

16 H 0.102512 -0.000297

17 H 0.109507 0.000679

**18 O -0.502232 0.402923**

19 O -0.643467 0.003872

20 H 0.281392 -0.000257

21 H 0.329494 -0.002074

--------------------------------------------------------

Sum of atomic charges = 0.000000

Sum of spin charges = 1.000000

**From Figure [5D].** Method: geometry opt+freq calculation: UB3LYP/6-31G(d,p), Euler-Maclaurin-Lebedev (EML) grid, containing 70 radial shells with 302 angular points per shell, neutral form, doublet. In blue the value of the spin-density for the phenoxyl oxygen and in red the C(4) atom.


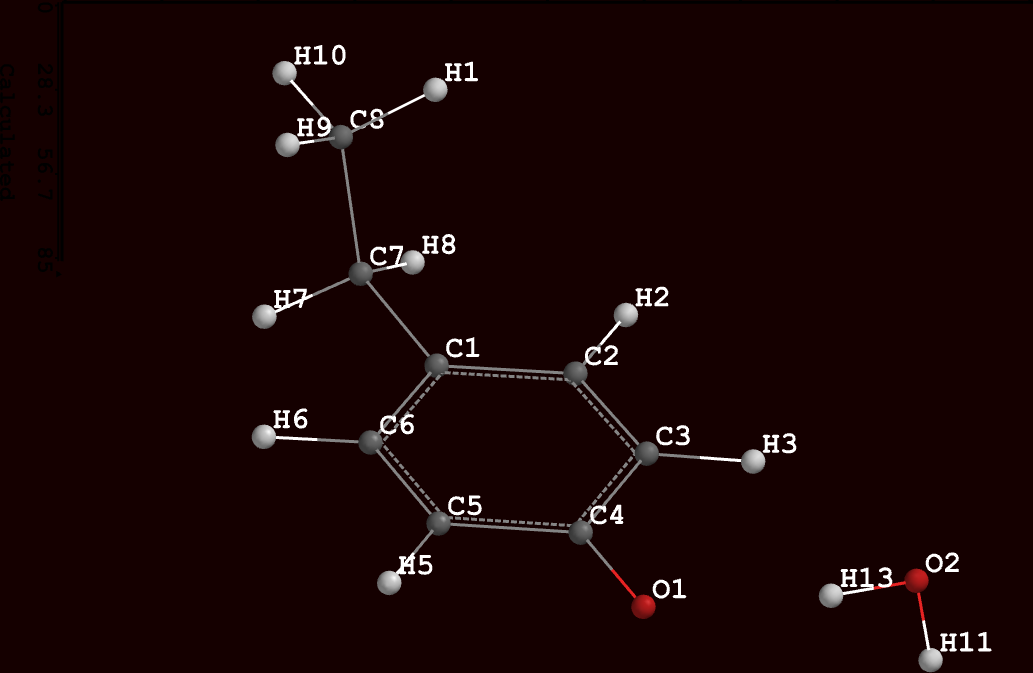


Natural Atomic Populations and Charges

Alpha spin Beta spin Spin

Atom Occupancy Occupancy Density Charge

------ ----------- ----------- ----------- ------------

C1 3.149687 2.804761 0.344926 0.045553

**C4 2.788008 2.829586 -0.041578 0.382407**

C2 3.069063 3.184483 -0.115420 -0.253546

C6 3.071726 3.181132 -0.109406 -0.252858

C5 3.235661 2.983470 0.252191 -0.219131

C3 3.239611 2.979742 0.259869 -0.219352

H2 0.378503 0.375397 0.003106 0.246100

H6 0.378945 0.375985 0.002960 0.245070

H5 0.370035 0.377670 -0.007635 0.252296

H3 0.361259 0.368663 -0.007404 0.270078

C7 3.236712 3.251674 -0.014961 -0.488386

H7 0.378102 0.372361 0.005740 0.249537

H8 0.377343 0.372758 0.004585 0.249899

C8 3.349037 3.328954 0.020083 -0.677990

H1 0.380741 0.381410 -0.000669 0.237849

H9 0.381543 0.382244 -0.000702 0.236213

H10 0.380435 0.377787 0.002649 0.241778

**O1 4.463724 4.059951 0.403773 -0.523674**

O2 4.491940 4.492966 -0.001026 -0.984906

H11 0.265881 0.265902 -0.000020 0.468217

H13 0.252046 0.253106 -0.001060 0.494848

Ground-State Mulliken Net Atomic Charges

Atom Charge (a.u.) Spin (a.u.)

--------------------------------------------------------

1 C 0.128750 0.396484

**2 C 0.379544 -0.071697**

3 C -0.115910 -0.158987

4 C -0.111991 -0.152036

5 C -0.093056 0.290866

6 C -0.116559 0.299150

7 H 0.096124 0.005206

8 H 0.095183 0.004939

9 H 0.114180 -0.012916

10 H 0.135570 -0.012444

11 C -0.249441 -0.026921

12 H 0.101930 0.006086

13 H 0.101907 0.004918

14 C -0.311169 0.020411

15 H 0.128751 -0.000281

16 H 0.126921 -0.000359

17 H 0.113149 0.005215

**18 O -0.504034 0.405983**

19 O -0.632435 -0.000725

20 H 0.284244 -0.000050

21 H 0.328340 -0.002843

--------------------------------------------------------

Sum of atomic charges = 0.000000

Sum of spin charges = 1.000000

**From Figure [5E].** Method: geometry opt+freq calculation: UB3LYP/6-31G(d,p), Euler-Maclaurin-Lebedev (EML) grid, containing 70 radial shells with 302 angular points per shell, neutral form, doublet. In blue the value of the spin-density for the phenoxyl oxygen and in red the C(4) atom.


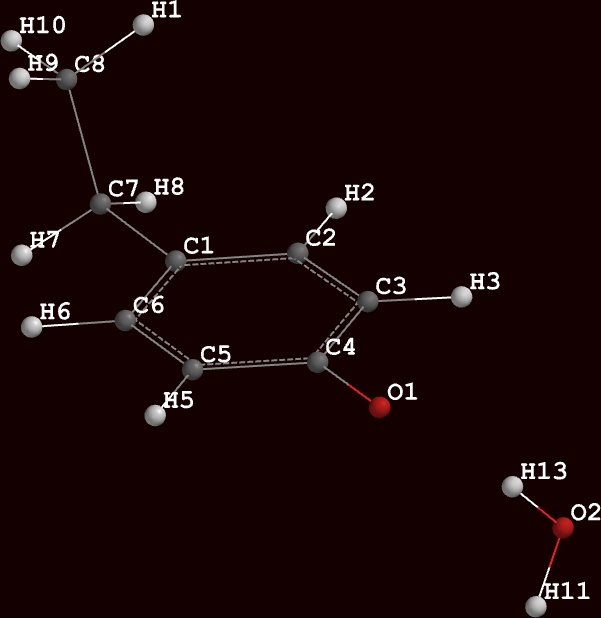


Natural Atomic Populations and Charges

Alpha spin Beta spin Spin

Atom Occupancy Occupancy Density Charge

------ ----------- ----------- ----------- ------------

C1 3.149428 2.804806 0.344623 0.045766

**C4 2.786533 2.829462 -0.042929 0.384006**

C2 3.069375 3.183734 -0.114360 -0.253109

C6 3.071003 3.181308 -0.110305 -0.252311

C5 3.235790 2.983473 0.252317 -0.219263

C3 3.235401 2.976256 0.259145 -0.211658

H2 0.378012 0.374925 0.003088 0.247063

H6 0.378836 0.375841 0.002995 0.245323

H5 0.369917 0.377561 -0.007644 0.252522

H3 0.364843 0.372609 -0.007766 0.262548

C7 3.236807 3.251745 -0.014938 -0.488552

H7 0.377787 0.372046 0.005741 0.250167

H8 0.377029 0.372436 0.004594 0.250535

C8 3.349022 3.328949 0.020073 -0.677970

H1 0.381008 0.381677 -0.000670 0.237315

H9 0.381557 0.382256 -0.000699 0.236187

H10 0.380225 0.377585 0.002640 0.242190

**O1 4.463874 4.062135 0.401739 -0.526009**

O2 4.491640 4.487890 0.003750 -0.979529

H11 0.267374 0.267539 -0.000165 0.465088

H13 0.254539 0.255767 -0.001228 0.489694

Ground-State Mulliken Net Atomic Charges

Atom Charge (a.u.) Spin (a.u.)

--------------------------------------------------------

1 C 0.129340 0.395701

**2 C 0.382017 -0.074280**

3 C -0.116824 -0.159549

4 C -0.112057 -0.152129

5 C -0.092307 0.291645

6 C -0.092803 0.299433

7 H 0.102154 0.005205

8 H 0.089110 0.004984

9 H 0.109712 -0.013008

10 H 0.138909 -0.012946

11 C -0.249283 -0.026919

12 H 0.098463 0.006101

13 H 0.106839 0.004894

14 C -0.311206 0.020207

15 H 0.131663 -0.000280

16 H 0.122389 -0.000338

17 H 0.111534 0.005202

**18 O -0.504676 0.404660**

19 O -0.654611 0.004031

20 H 0.282677 -0.000176

21 H 0.328960 -0.002438

--------------------------------------------------------

Sum of atomic charges = 0.000000

Sum of spin charges = 1.000000

**From Figure 5F.** Method: geometry opt+freq calculation: UB3LYP/6-31G(d,p), Euler-Maclaurin-Lebedev (EML) grid, containing 70 radial shells with 302 angular points per shell, neutral form, doublet. In blue the value of the spin-density for the phenoxyl oxygen and in red the C(4) atom.


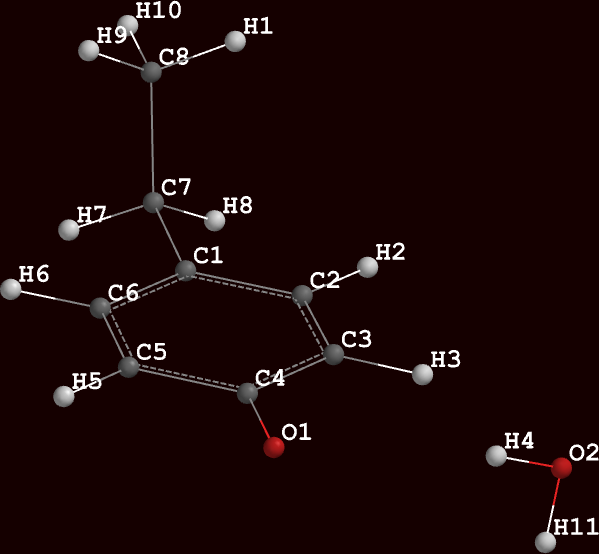


Natural Atomic Populations and Charges

Alpha spin Beta spin Spin

Atom Occupancy Occupancy Density Charge

------ ----------- ----------- ----------- ------------

C1 3.151452 2.808013 0.343439 0.040535

**C4 2.784605 2.840774 -0.056169 0.374622**

C2 3.068538 3.185877 -0.117339 -0.254415

C6 3.070627 3.183738 -0.113112 -0.254365

C5 3.239975 2.981041 0.258933 -0.221016

C3 3.242436 2.979960 0.262475 -0.222396

H2 0.379129 0.375960 0.003169 0.244911

H6 0.379656 0.376594 0.003062 0.243750

H5 0.370884 0.378694 -0.007810 0.250422

H3 0.364998 0.372487 -0.007488 0.262515

C7 3.236311 3.251425 -0.015114 -0.487736

H7 0.378340 0.373169 0.005172 0.248491

H8 0.377860 0.372752 0.005108 0.249387

C8 3.348886 3.329133 0.019754 -0.678019

H1 0.381082 0.381754 -0.000672 0.237164

H9 0.381663 0.382339 -0.000676 0.235998

H10 0.380836 0.378260 0.002576 0.240904

**O1 4.465691 4.050103 0.415588 -0.515794**

H4 0.254171 0.254633 -0.000461 0.491196

O2 4.481905 4.482310 -0.000406 -0.964215

H11 0.260956 0.260985 -0.000029 0.478059

Ground-State Mulliken Net Atomic Charges

Atom Charge (a.u.) Spin (a.u.)

--------------------------------------------------------

1 C 0.127561 0.395105

**2 C 0.375393 -0.088982**

3 C -0.116938 -0.161294

4 C -0.112589 -0.156433

5 C -0.097957 0.299304

6 C -0.118168 0.303286

7 H 0.094351 0.005346

8 H 0.092294 0.005142

9 H 0.110712 -0.013314

10 H 0.122228 -0.012888

11 C -0.248427 -0.026992

12 H 0.100212 0.005497

13 H 0.100768 0.005441

14 C -0.310863 0.020030

15 H 0.127558 -0.000311

16 H 0.126420 -0.000307

17 H 0.111426 0.005102

**18 O -0.502717 0.417780**

19 H 0.321488 -0.001209

20 O -0.600217 -0.000238

21 H 0.297466 -0.000064

--------------------------------------------------------

Sum of atomic charges = 0.000000

Sum of spin charges = 1.000000

**From Figure 5G.** Method: geometry opt+freq calculation: UB3LYP/6-31G(d,p), Euler-Maclaurin-Lebedev (EML) grid, containing 70 radial shells with 302 angular points per shell, neutral form, doublet. In blue the value of the spin-density for the phenoxyl oxygen and in red the C(4) atom.


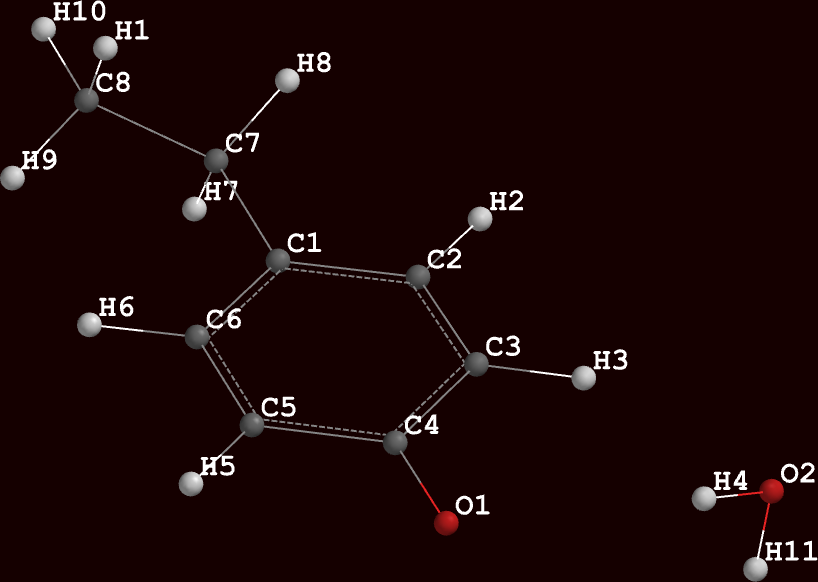


Natural Atomic Populations and Charges

Alpha spin Beta spin Spin

Atom Occupancy Occupancy Density Charge

------ ----------- ----------- ----------- ------------

C1 3.151293 2.808011 0.343282 0.040696

**C4 2.784832 2.840517 -0.055685 0.374651**

C2 3.067155 3.184466 -0.117311 -0.251620

C6 3.072138 3.185130 -0.112991 -0.257268

C5 3.239501 2.979319 0.260182 -0.218820

C3 3.241937 2.981102 0.260836 -0.223039

H2 0.379160 0.375983 0.003177 0.244858

H6 0.380400 0.377378 0.003023 0.242222

H5 0.371098 0.378958 -0.007860 0.249944

H3 0.365041 0.372505 -0.007464 0.262453

C7 3.236480 3.250803 -0.014323 -0.487283

H7 0.380704 0.360750 0.019955 0.258546

H8 0.377209 0.372161 0.005048 0.250630

C8 3.345594 3.339919 0.005675 -0.685513

H1 0.380260 0.380291 -0.000031 0.239450

H9 0.386208 0.386419 -0.000211 0.227373

H10 0.378110 0.377588 0.000521 0.244302

**O1 4.465911 4.050843 0.415068 -0.516754**

H4 0.254246 0.254697 -0.000452 0.491057

O2 4.481893 4.482299 -0.000406 -0.964192

H11 0.260829 0.260862 -0.000032 0.478309

Ground-State Mulliken Net Atomic Charges

Atom Charge (a.u.) Spin (a.u.)

--------------------------------------------------------

1 C 0.131189 0.397822

**2 C 0.377044 -0.087260**

3 C -0.122971 -0.161762

4 C -0.104813 -0.156646

5 C -0.107357 0.300238

6 C -0.118859 0.301236

7 H 0.096162 0.005335

8 H 0.090233 0.005186

9 H 0.107966 -0.013379

10 H 0.122550 -0.012809

11 C -0.249236 -0.028787

12 H 0.112236 0.022585

13 H 0.125155 0.006066

14 C -0.326108 0.006160

15 H 0.139399 0.000486

16 H 0.105727 -0.000392

17 H 0.108641 0.001263

**18 O -0.506000 0.416150**

19 H 0.321036 -0.001180

20 O -0.599506 -0.000236

21 H 0.297512 -0.000077

--------------------------------------------------------

Sum of atomic charges = 0.000000

Sum of spin charges = 1.000000

**From Figure 5H.** Method: geometry opt+freq calculation: UB3LYP/6-31G(d,p), Euler-Maclaurin-Lebedev (EML) grid, containing 70 radial shells with 302 angular points per shell, neutral form, doublet. In blue the value of the spin-density for the phenoxyl oxygen and in red the C(4) atom.

**
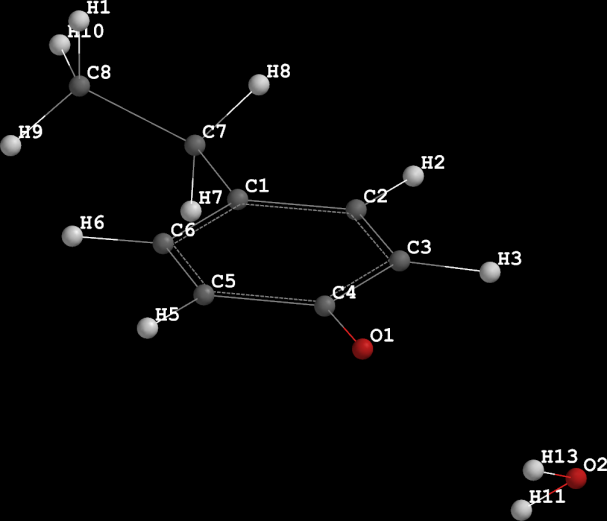
**

**Alpha spin Beta spin Spin**

**Atom Occupancy Occupancy Density Charge**

**------ ----------- ----------- ----------- ------------**

**C1 3.150613 2.806914 0.343699 0.042472**

**C4 2.785953 2.843137 -0.057184 0.370910**

**C2 3.068536 3.184600 -0.116063 -0.253136**

**C6 3.072547 3.186095 -0.113548 -0.258642**

**C5 3.242408 2.977288 0.265120 -0.219696**

**C3 3.238940 2.984560 0.254381 -0.223500**

**H2 0.379249 0.376108 0.003141 0.244643**

**H6 0.379179 0.376138 0.003041 0.244683**

**H5 0.370944 0.378918 -0.007975 0.250138**

**H3 0.363906 0.371422 -0.007516 0.264672**

**C7 3.237643 3.251737 -0.014094 -0.489380**

**H7 0.380488 0.359659 0.020828 0.259853**

**H8 0.377520 0.372134 0.005387 0.250346**

**C8 3.347338 3.342557 0.004781 -0.689895**

**H1 0.380564 0.380358 0.000206 0.239077**

**H9 0.384098 0.384257 -0.000159 0.231646**

**H10 0.377099 0.376939 0.000160 0.245961**

**O1 4.463063 4.047769 0.415294 -0.510831**

**O2 4.481123 4.480310 0.000813 -0.961434**

**H11 0.263270 0.263352 -0.000082 0.473378**

**H13 0.255519 0.255748 -0.000230 0.488733**

**Total Charge = 0.00**

**Ground-State Mulliken Net Atomic Charges**

**Atom Charge (a.u.) Spin (a.u.)**

**--------------------------------------------------------**

**1 C 0.133496 0.397899**

**2 C 0.369944 -0.092208**

**3 C -0.124142 -0.161868**

**4 C -0.112412 -0.158166**

**5 C -0.103763 0.307507**

**6 C -0.109455 0.295533**

**7 H 0.097823 0.005351**

**8 H 0.088524 0.005196**

**9 H 0.107724 -0.013686**

**10 H 0.136389 -0.012623**

**11 C -0.247742 -0.028938**

**12 H 0.111939 0.023313**

**13 H 0.124853 0.006416**

**14 C -0.325619 0.005212**

**15 H 0.136772 0.000836**

**16 H 0.102160 -0.000300**

**17 H 0.107804 0.000651**

**18 O -0.494128 0.419419**

**19 O -0.614120 0.000994**

**20 H 0.293785 0.000030**

**21 H 0.320168 -0.000566**

**--------------------------------------------------------**

**Sum of atomic charges = 0.000000**

**Sum of spin charges = 1.000000**

**From Figure 5I:** Geometry optimization followed by frequency calculation of the ethylphenoxyl radical under constraint angle (θ = 30°) at UB3LYP/6-31G(d,p) with Euler-Maclaurin-Lebedev (EML) grid (70,302), obtained after placing a positive charge (Li^+^) at 2.60Å on the phenoxyl plane. On the left, atom numbering, on the right, Mulliken spin density (0.002 isovalue). In the table below, in blue the value of the spin-density for the phenoxyl oxygen and in red the C(4) atom.


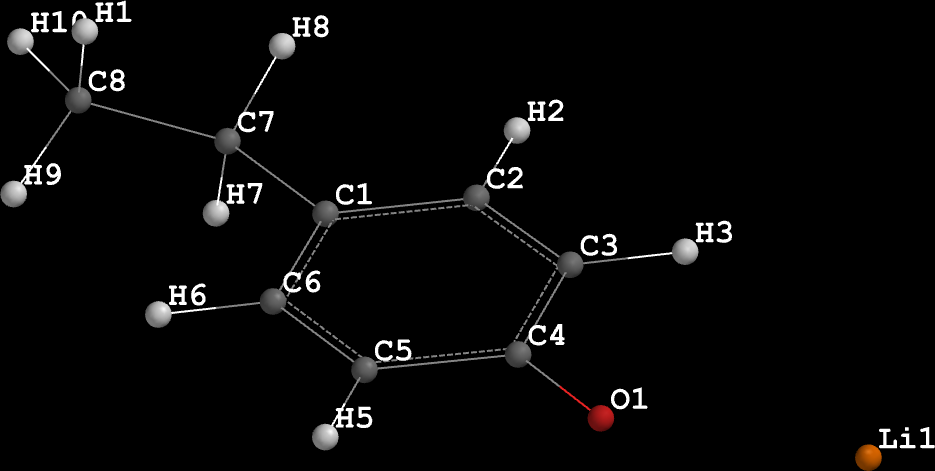


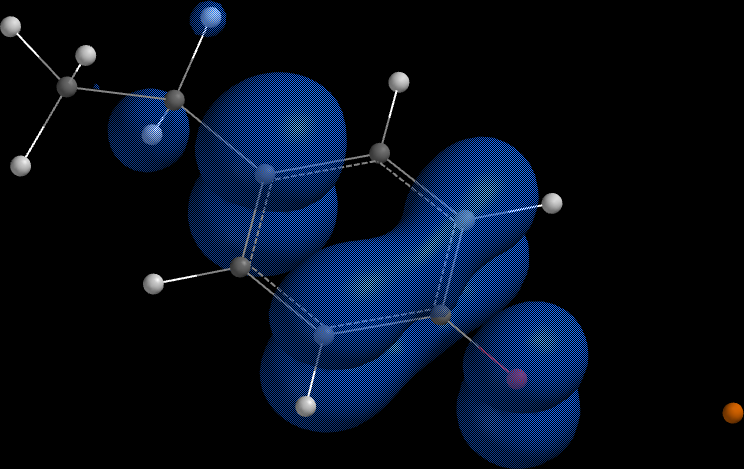


Natural Atomic Populations and Charges

Alpha spin Beta spin Spin

Atom Occupancy Occupancy Density Charge

------ ----------- ----------- ----------- ------------

C1 3.133430 2.778673 0.354758 0.087897

C4 2.807153 2.772772 0.034381 0.420076

C2 3.075920 3.148880 -0.072959 -0.224800

C6 3.073098 3.166034 -0.092936 -0.239132

C5 3.220587 2.991891 0.228696 -0.212478

C3 3.221793 3.020162 0.201630 -0.241955

H2 0.370755 0.368943 0.001813 0.260302

H6 0.369241 0.366898 0.002343 0.263862

H5 0.364510 0.371434 -0.006924 0.264056

H3 0.377918 0.384255 -0.006337 0.237827

C7 3.245519 3.255370 -0.009852 -0.500889

H7 0.372956 0.348370 0.024586 0.278673

H8 0.372564 0.366342 0.006222 0.261094

C8 3.350353 3.344857 0.005496 -0.695211

H1 0.378160 0.377719 0.000441 0.244120

H9 0.381181 0.381259 -0.000078 0.237560

H10 0.369533 0.369326 0.000207 0.261141

O1 4.495282 4.165113 0.330169 -0.660396

Li1 1.020045 1.021701 -0.001657 0.958254

Total Charge = 1.00

Ground-State Mulliken Net Atomic Charges

Atom Charge (a.u.) Spin (a.u.)

--------------------------------------------------------

1 C 0.136590 0.406993

2 C 0.420487 0.022077

3 C -0.103707 -0.108037

4 C -0.097181 -0.132652

5 C -0.097882 0.257997

6 C -0.105864 0.227175

7 H 0.125925 0.002988

8 H 0.128899 0.003910

9 H 0.137539 -0.011544

10 H 0.099236 -0.010800

11 C -0.259869 -0.026112

12 H 0.156294 0.028321

13 H 0.133696 0.007599

14 C -0.334502 0.005972

15 H 0.128903 0.001204

16 H 0.123962 -0.000166

17 H 0.137285 0.000703

18 O -0.564838 0.328913

19 Li 0.835025 -0.004540

--------------------------------------------------------

Sum of atomic charges = 1.000000

Sum of spin charges = 1.000000

**Figure S1.** Method: geometry opt without constraint: UB3LYP/6-31G(d,p), SG-1 grid, neutral form, doublet. In blue the value of the spin-density for the phenoxyl oxygen and in red the C(4) atom.

Ground-State Mulliken Net Atomic Charges

Atom Charge (a.u.) Spin (a.u.)

--------------------------------------------------------

1 C 0.127238 0.395059

**2 C 0.380249 -0.106695**

3 C -0.112175 -0.161778

4 C -0.112175 -0.161778

5 C -0.103332 0.307489

6 C -0.103332 0.307489

7 H 0.092639 0.005394

8 H 0.092639 0.005395

9 H 0.108551 -0.013692

10 H 0.108551 -0.013692

11 C -0.249186 -0.026987

12 H 0.111099 0.005441

13 H 0.111099 0.005441

14 C -0.311237 0.019452

15 H 0.115633 -0.000263

16 H 0.115633 -0.000263

17 H 0.112426 0.004970

**18 O -0.484319 0.429016**

--------------------------------------------------------

Sum of atomic charges = 0.000000

Sum of spin charges = 1.000000


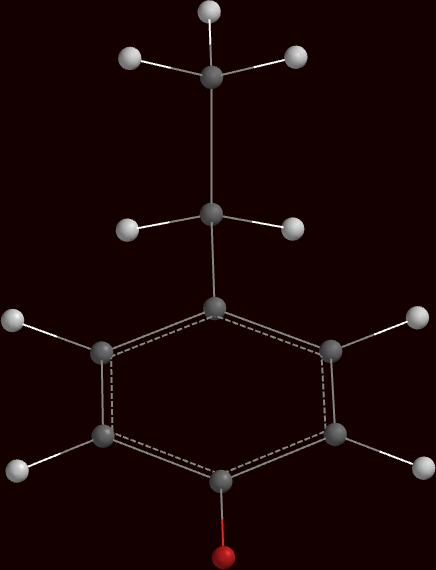


**PART II: Vibrational analyses for the ethylphenoxyl radical around the phenoxyl stretching vibration (low-frequency range).**

**From Figure 5A. The Mode: 35, corresponds to the C-O stretching vibration.**

**Note: also Mode: 36 contains part of the C-O stretching vibration.**

**********************************************************************

** **

** VIBRATIONAL ANALYSIS **

** -------------------- **

** **

** VIBRATIONAL FREQUENCIES (CM**-1) AND NORMAL MODES **

** FORCE CONSTANTS (mDYN/ANGSTROM) AND REDUCED MASSES (AMU) **

** INFRARED INTENSITIES (KM/MOL) **

** RAMAN SCATTERING ACTIVITIES (A**4/AMU) AND DEPOLARIZATION RATIOS **

** **

**********************************************************************

Mode: 22 23 24

Frequency: 1067.70 1092.99 1131.84

Force Cnst: 1.2484 1.1358 1.0118

Red. Mass: 1.8586 1.6137 1.3405

IR Active: YES YES YES

IR Intens: 5.778 2.581 4.934

Raman Active: YES YES YES

Raman Intens: 2.554 1.190 1.458

Depolar: 0.232 0.269 0.498

Mode: 25 26 27

Frequency: 1174.08 1214.72 1282.59

Force Cnst: 0.9843 2.0975 2.1784

Red. Mass: 1.2120 2.4127 2.2475

IR Active: YES YES YES

IR Intens: 1.766 5.133 4.293

Raman Active: YES YES YES

Raman Intens: 4.875 15.851 1.958

Depolar: 0.639 0.186 0.703

Mode: 28 29 30

Frequency: 1286.23 1310.13 1385.62

Force Cnst: 1.3483 2.0511 1.8366

Red. Mass: 1.3833 2.0282 1.6236

IR Active: YES YES YES

IR Intens: 0.898 4.744 9.093

Raman Active: YES YES YES

Raman Intens: 8.325 22.611 9.282

Depolar: 0.639 0.404 0.407

Mode: 31 32 33

Frequency: 1427.85 1441.06 1457.70

Force Cnst: 1.5480 3.2342 3.1150

Red. Mass: 1.2887 2.6433 2.4881

IR Active: YES YES YES

IR Intens: 1.157 0.830 4.085

Raman Active: YES YES YES

Raman Intens: 6.641 2.307 8.688

Depolar: 0.746 0.324 0.738

Mode: 34 35 36

Frequency: 1482.38 1510.24 1514.45

Force Cnst: 1.4444 3.0399 1.7921

Red. Mass: 1.1156 2.2622 1.3262

IR Active: YES YES YES

IR Intens: 3.072 12.508 12.927

Raman Active: YES YES YES

Raman Intens: 25.630 54.443 34.202

Depolar: 0.581 0.270 0.351

Mode: 37 38 39

Frequency: 1520.08 1524.90 1614.98

Force Cnst: 2.3807 2.1698 7.7908

Red. Mass: 1.7487 1.5837 5.0699

IR Active: YES YES YES

IR Intens: 5.055 3.681 80.093

Raman Active: YES YES YES

Raman Intens: 10.032 10.178 56.108

Depolar: 0.678 0.558 0.737

**From Figure 5B. The Mode: 44, corresponds to the C-O stretching vibration**

**********************************************************************

** **

** VIBRATIONAL ANALYSIS **

** -------------------- **

** **

** VIBRATIONAL FREQUENCIES (CM**-1) AND NORMAL MODES **

** FORCE CONSTANTS (mDYN/ANGSTROM) AND REDUCED MASSES (AMU) **

** INFRARED INTENSITIES (KM/MOL) **

** RAMAN SCATTERING ACTIVITIES (A**4/AMU) AND DEPOLARIZATION RATIOS **

** **

**********************************************************************

Mode: 28 29 30

Frequency: 1069.18 1092.16 1138.82

Force Cnst: 1.2962 1.1282 1.0305

Red. Mass: 1.9245 1.6053 1.3486

IR Active: YES YES YES

IR Intens: 5.807 3.248 2.421

Raman Active: YES YES YES

Raman Intens: 2.334 0.937 2.456

Depolar: 0.294 0.309 0.496

Mode: 31 32 33

Frequency: 1183.28 1217.85 1285.84

Force Cnst: 1.0401 1.9459 1.3308

Red. Mass: 1.2608 2.2268 1.3661

IR Active: YES YES YES

IR Intens: 4.325 3.036 1.386

Raman Active: YES YES YES

Raman Intens: 6.417 27.243 11.777

Depolar: 0.634 0.213 0.559

Mode: 34 35 36

Frequency: 1301.41 1310.71 1388.14

Force Cnst: 2.0710 2.1952 1.8362

Red. Mass: 2.0754 2.1687 1.6173

IR Active: YES YES YES

IR Intens: 4.626 4.114 10.388

Raman Active: YES YES YES

Raman Intens: 9.808 21.471 12.366

Depolar: 0.465 0.414 0.357

Mode: 37 38 39

Frequency: 1428.32 1439.81 1458.39

Force Cnst: 1.5364 3.1868 3.2725

Red. Mass: 1.2782 2.6091 2.6115

IR Active: YES YES YES

IR Intens: 0.964 0.137 6.786

Raman Active: YES YES YES

Raman Intens: 7.013 8.730 7.091

Depolar: 0.718 0.350 0.710

Mode: 40 41 42

Frequency: 1480.47 1513.01 1519.61

Force Cnst: 1.4344 1.4561 1.7105

Red. Mass: 1.1108 1.0796 1.2572

IR Active: YES YES YES

IR Intens: 4.046 7.277 8.602

Raman Active: YES YES YES

Raman Intens: 30.577 20.644 23.975

Depolar: 0.523 0.688 0.489

Mode: 43 44 45

Frequency: 1524.55 1529.06 1617.40

Force Cnst: 3.2159 5.0075 7.7750

Red. Mass: 2.3484 3.6351 5.0445

IR Active: YES YES YES

IR Intens: 3.646 63.456 81.604

Raman Active: YES YES YES

Raman Intens: 5.098 97.950 47.029

Depolar: 0.349 0.268 0.749

**From Figure 5C. The Mode: 43 and 44, contains both large contributions**

**to the C-O stretching vibration.**

**********************************************************************

** **

** VIBRATIONAL ANALYSIS **

** -------------------- **

** **

** VIBRATIONAL FREQUENCIES (CM**-1) AND NORMAL MODES **

** FORCE CONSTANTS (mDYN/ANGSTROM) AND REDUCED MASSES (AMU) **

** INFRARED INTENSITIES (KM/MOL) **

** RAMAN SCATTERING ACTIVITIES (A**4/AMU) AND DEPOLARIZATION RATIOS **

** **

**********************************************************************

Mode: 28 29 30

Frequency: 1069.83 1092.63 1137.77

Force Cnst: 1.2786 1.1263 1.0268

Red. Mass: 1.8961 1.6012 1.3462

IR Active: YES YES YES

IR Intens: 6.009 2.955 4.218

Raman Active: YES YES YES

Raman Intens: 2.463 0.967 1.660

Depolar: 0.232 0.287 0.491

Mode: 31 32 33

Frequency: 1181.81 1217.32 1287.09

Force Cnst: 1.0075 2.0322 1.3245

Red. Mass: 1.2243 2.3276 1.3570

IR Active: YES YES YES

IR Intens: 2.659 5.389 1.307

Raman Active: YES YES YES

Raman Intens: 6.499 21.965 11.355

Depolar: 0.591 0.196 0.573

Mode: 34 35 36

Frequency: 1294.90 1312.20 1388.23

Force Cnst: 2.0494 2.2700 1.8415

Red. Mass: 2.0745 2.2375 1.6218

IR Active: YES YES YES

IR Intens: 3.567 4.451 9.370

Raman Active: YES YES YES

Raman Intens: 2.946 22.697 10.856

Depolar: 0.554 0.400 0.373

Mode: 37 38 39

Frequency: 1429.68 1441.49 1459.32

Force Cnst: 1.5470 3.1314 3.1357

Red. Mass: 1.2846 2.5578 2.4991

IR Active: YES YES YES

IR Intens: 0.905 0.186 5.566

Raman Active: YES YES YES

Raman Intens: 7.055 6.612 8.526

Depolar: 0.727 0.336 0.748

Mode: 40 41 42

Frequency: 1480.66 1513.97 1521.52

Force Cnst: 1.4378 1.4524 2.6304

Red. Mass: 1.1131 1.0755 1.9285

IR Active: YES YES YES

IR Intens: 4.012 5.326 6.624

Raman Active: YES YES YES

Raman Intens: 28.323 17.821 11.022

Depolar: 0.536 0.685 0.570

Mode: 43 44 45

Frequency: 1525.52 1526.81 1617.06

Force Cnst: 2.6852 3.1436 7.7235

Red. Mass: 1.9584 2.2888 5.0132

IR Active: YES YES YES

IR Intens: 29.734 24.534 90.344

Raman Active: YES YES YES

Raman Intens: 77.659 64.235 47.325

Depolar: 0.303 0.243 0.750

**From Figure 5D. The Mode: 44, corresponds to the C-O stretching vibration.**

**********************************************************************

** **

** VIBRATIONAL ANALYSIS **

** -------------------- **

** **

** VIBRATIONAL FREQUENCIES (CM**-1) AND NORMAL MODES **

** FORCE CONSTANTS (mDYN/ANGSTROM) AND REDUCED MASSES (AMU) **

** INFRARED INTENSITIES (KM/MOL) **

** RAMAN SCATTERING ACTIVITIES (A**4/AMU) AND DEPOLARIZATION RATIOS **

** **

**********************************************************************

Mode: 28 29 30

Frequency: 1064.99 1073.26 1136.80

Force Cnst: 1.0058 1.2436 0.9810

Red. Mass: 1.5051 1.8325 1.2884

IR Active: YES YES YES

IR Intens: 4.024 19.975 2.595

Raman Active: YES YES YES

Raman Intens: 2.413 20.390 1.120

Depolar: 0.688 0.169 0.693

Mode: 31 32 33

Frequency: 1182.74 1229.76 1267.88

Force Cnst: 0.9920 2.2303 1.4285

Red. Mass: 1.2036 2.5030 1.5083

IR Active: YES YES YES

IR Intens: 2.732 0.663 0.702

Raman Active: YES YES YES

Raman Intens: 8.344 28.023 2.060

Depolar: 0.539 0.205 0.698

Mode: 34 35 36

Frequency: 1298.58 1349.69 1354.11

Force Cnst: 2.0356 1.9630 1.6615

Red. Mass: 2.0488 1.8289 1.5379

IR Active: YES YES YES

IR Intens: 5.016 0.920 0.726

Raman Active: YES YES YES

Raman Intens: 8.744 11.700 56.399

Depolar: 0.529 0.651 0.404

Mode: 37 38 39

Frequency: 1424.81 1439.78 1455.58

Force Cnst: 1.4741 3.1933 3.5377

Red. Mass: 1.2325 2.6146 2.8340

IR Active: YES YES YES

IR Intens: 2.872 0.650 6.392

Raman Active: YES YES YES

Raman Intens: 5.798 13.295 12.454

Depolar: 0.617 0.349 0.674

Mode: 40 41 42

Frequency: 1504.53 1511.98 1521.81

Force Cnst: 1.4311 1.4029 3.6297

Red. Mass: 1.0731 1.0416 2.6601

IR Active: YES YES YES

IR Intens: 1.569 8.691 23.161

Raman Active: YES YES YES

Raman Intens: 28.081 20.157 47.153

Depolar: 0.750 0.747 0.274

Mode: 43 44 45

Frequency: 1523.72 1528.68 1617.43

Force Cnst: 1.9175 4.1441 7.9098

Red. Mass: 1.4018 3.0099 5.1318

IR Active: YES YES YES

IR Intens: 1.913 44.280 75.324

Raman Active: YES YES YES

Raman Intens: 3.203 65.489 51.419

Depolar: 0.430 0.260 0.744

**From Figure 5E. The Mode: 42, corresponds to the C-O stretching vibration.**

**********************************************************************

** **

** VIBRATIONAL ANALYSIS **

** -------------------- **

** **

** VIBRATIONAL FREQUENCIES (CM**-1) AND NORMAL MODES **

** FORCE CONSTANTS (mDYN/ANGSTROM) AND REDUCED MASSES (AMU) **

** INFRARED INTENSITIES (KM/MOL) **

** RAMAN SCATTERING ACTIVITIES (A**4/AMU) AND DEPOLARIZATION RATIOS **

** **

**********************************************************************

Mode: 28 29 30

Frequency: 1062.71 1071.06 1133.90

Force Cnst: 0.9930 1.2489 0.9690

Red. Mass: 1.4923 1.8479 1.2792

IR Active: YES YES YES

IR Intens: 4.348 21.219 4.145

Raman Active: YES YES YES

Raman Intens: 2.058 19.586 0.535

Depolar: 0.710 0.170 0.745

Mode: 31 32 33

Frequency: 1178.32 1228.97 1265.93

Force Cnst: 0.9678 2.2724 1.4112

Red. Mass: 1.1830 2.5536 1.4946

IR Active: YES YES YES

IR Intens: 1.363 1.798 0.714

Raman Active: YES YES YES

Raman Intens: 8.057 22.294 2.393

Depolar: 0.493 0.193 0.701

Mode: 34 35 36

Frequency: 1291.30 1347.56 1351.50

Force Cnst: 2.0030 1.9947 1.6857

Red. Mass: 2.0388 1.8643 1.5664

IR Active: YES YES YES

IR Intens: 2.894 1.577 0.625

Raman Active: YES YES YES

Raman Intens: 2.239 16.423 52.520

Depolar: 0.696 0.573 0.418

Mode: 37 38 39

Frequency: 1420.16 1439.22 1456.32

Force Cnst: 1.4491 3.2948 3.4713

Red. Mass: 1.2195 2.6998 2.7780

IR Active: YES YES YES

IR Intens: 2.868 1.193 4.841

Raman Active: YES YES YES

Raman Intens: 6.398 15.659 12.304

Depolar: 0.622 0.275 0.733

Mode: 40 41 42

Frequency: 1501.99 1509.49 1518.58

Force Cnst: 1.4304 1.3993 3.6746

Red. Mass: 1.0761 1.0423 2.7045

IR Active: YES YES YES

IR Intens: 1.461 8.735 38.504

Raman Active: YES YES YES

Raman Intens: 25.671 19.897 130.163

Depolar: 0.743 0.750 0.253

Mode: 43 44 45

Frequency: 1523.24 1523.64 1616.51

Force Cnst: 3.4612 2.1350 7.8701

Red. Mass: 2.5318 1.5609 5.1118

IR Active: YES YES YES

IR Intens: 3.172 8.896 83.913

Raman Active: YES YES YES

Raman Intens: 6.280 27.382 52.942

Depolar: 0.337 0.260 0.744

**From Figure 5F. The Mode: 42, corresponds to the C-O stretching vibration.**

**********************************************************************

** **

** VIBRATIONAL ANALYSIS **

** -------------------- **

** **

** VIBRATIONAL FREQUENCIES (CM**-1) AND NORMAL MODES **

** FORCE CONSTANTS (mDYN/ANGSTROM) AND REDUCED MASSES (AMU) **

** INFRARED INTENSITIES (KM/MOL) **

** RAMAN SCATTERING ACTIVITIES (A**4/AMU) AND DEPOLARIZATION RATIOS **

** **

**********************************************************************

Mode: 28 29 30

Frequency: 1063.60 1072.07 1136.49

Force Cnst: 0.9970 1.2538 0.9830

Red. Mass: 1.4959 1.8515 1.2918

IR Active: YES YES YES

IR Intens: 2.714 19.467 2.487

Raman Active: YES YES YES

Raman Intens: 2.530 18.410 1.372

Depolar: 0.730 0.159 0.731

Mode: 31 32 33

Frequency: 1182.13 1230.65 1266.05

Force Cnst: 0.9960 2.2250 1.4446

Red. Mass: 1.2096 2.4935 1.5296

IR Active: YES YES YES

IR Intens: 2.262 0.707 0.779

Raman Active: YES YES YES

Raman Intens: 5.833 25.547 1.912

Depolar: 0.521 0.184 0.726

Mode: 34 35 36

Frequency: 1295.01 1348.47 1351.84

Force Cnst: 2.0402 1.9810 1.6276

Red. Mass: 2.0648 1.8491 1.5117

IR Active: YES YES YES

IR Intens: 5.995 0.751 0.803

Raman Active: YES YES YES

Raman Intens: 8.624 8.326 55.488

Depolar: 0.556 0.727 0.408

Mode: 37 38 39

Frequency: 1421.57 1441.48 1454.56

Force Cnst: 1.4518 3.3524 3.4842

Red. Mass: 1.2193 2.7383 2.7951

IR Active: YES YES YES

IR Intens: 2.916 0.293 5.282

Raman Active: YES YES YES

Raman Intens: 6.067 10.966 11.927

Depolar: 0.626 0.313 0.716

Mode: 40 41 42

Frequency: 1501.57 1510.50 1518.26

Force Cnst: 1.4305 1.4010 3.9314

Red. Mass: 1.0768 1.0422 2.8947

IR Active: YES YES YES

IR Intens: 1.605 8.444 27.450

Raman Active: YES YES YES

Raman Intens: 27.216 19.952 62.931

Depolar: 0.749 0.749 0.254

Mode: 43 44 45

Frequency: 1523.01 1526.62 1615.64

Force Cnst: 1.6749 4.9691 7.8772

Red. Mass: 1.2256 3.6189 5.1219

IR Active: YES YES YES

IR Intens: 4.669 11.548 75.967

Raman Active: YES YES YES

Raman Intens: 11.191 17.632 56.697

Depolar: 0.327 0.288 0.730

**From Figure 5G. The Mode: 42, corresponds to the C-O stretching vibration.**

**********************************************************************

** **

** VIBRATIONAL ANALYSIS **

** -------------------- **

** **

** VIBRATIONAL FREQUENCIES (CM**-1) AND NORMAL MODES **

** FORCE CONSTANTS (mDYN/ANGSTROM) AND REDUCED MASSES (AMU) **

** INFRARED INTENSITIES (KM/MOL) **

** RAMAN SCATTERING ACTIVITIES (A**4/AMU) AND DEPOLARIZATION RATIOS **

** **

**********************************************************************

Mode: 28 29 30

Frequency: 1064.92 1088.81 1146.54

Force Cnst: 1.2144 1.0966 1.0457

Red. Mass: 1.8174 1.5700 1.3501

IR Active: YES YES YES

IR Intens: 6.294 1.865 1.850

Raman Active: YES YES YES

Raman Intens: 3.376 2.070 2.820

Depolar: 0.227 0.282 0.408

Mode: 31 32 33

Frequency: 1185.28 1222.00 1274.13

Force Cnst: 1.0315 1.9372 1.2254

Red. Mass: 1.2462 2.2018 1.2812

IR Active: YES YES YES

IR Intens: 2.761 2.403 0.857

Raman Active: YES YES YES

Raman Intens: 5.120 20.967 15.733

Depolar: 0.595 0.185 0.541

Mode: 34 35 36

Frequency: 1296.25 1309.40 1374.64

Force Cnst: 2.6074 2.0298 1.8722

Red. Mass: 2.6337 2.0094 1.6816

IR Active: YES YES YES

IR Intens: 6.428 3.394 6.683

Raman Active: YES YES YES

Raman Intens: 13.959 8.624 13.175

Depolar: 0.464 0.441 0.336

Mode: 37 38 39

Frequency: 1424.48 1443.36 1460.91

Force Cnst: 1.5454 3.3987 2.9303

Red. Mass: 1.2926 2.7689 2.3303

IR Active: YES YES YES

IR Intens: 1.196 0.554 5.018

Raman Active: YES YES YES

Raman Intens: 6.197 7.658 8.937

Depolar: 0.668 0.358 0.708

Mode: 40 41 42

Frequency: 1478.95 1513.69 1518.63

Force Cnst: 1.4404 1.7668 2.4850

Red. Mass: 1.1177 1.3087 1.8288

IR Active: YES YES YES

IR Intens: 2.690 10.373 25.277

Raman Active: YES YES YES

Raman Intens: 30.974 21.304 45.496

Depolar: 0.542 0.468 0.304

Mode: 43 44 45

Frequency: 1525.56 1530.69 1618.24

Force Cnst: 4.0091 1.7752 7.5221

Red. Mass: 2.9237 1.2860 4.8754

IR Active: YES YES YES

IR Intens: 17.830 4.103 84.950

Raman Active: YES YES YES

Raman Intens: 38.508 9.749 53.524

Depolar: 0.273 0.746 0.738

**From Figure 5H. The Mode: 42, corresponds to the C-O stretching vibration.**

**********************************************************************

** **

** VIBRATIONAL ANALYSIS **

** -------------------- **

** **

** VIBRATIONAL FREQUENCIES (CM**-1) AND NORMAL MODES **

** FORCE CONSTANTS (mDYN/ANGSTROM) AND REDUCED MASSES (AMU) **

** INFRARED INTENSITIES (KM/MOL) **

** RAMAN SCATTERING ACTIVITIES (A**4/AMU) AND DEPOLARIZATION RATIOS **

** **

**********************************************************************

Mode: 28 29 30

Frequency: 1069.46 1092.98 1136.47

Force Cnst: 1.2747 1.1287 1.0269

Red. Mass: 1.8916 1.6036 1.3494

IR Active: YES YES YES

IR Intens: 5.626 2.724 4.165

Raman Active: YES YES YES

Raman Intens: 2.366 1.029 2.118

Depolar: 0.250 0.284 0.477

Mode: 31 32 33

Frequency: 1179.61 1216.89 1287.00

Force Cnst: 1.0054 2.0519 1.4402

Red. Mass: 1.2263 2.3518 1.4757

IR Active: YES YES YES

IR Intens: 2.233 4.914 1.923

Raman Active: YES YES YES

Raman Intens: 4.752 19.758 8.671

Depolar: 0.600 0.176 0.637

Mode: 34 35 36

Frequency: 1290.82 1311.14 1387.60

Force Cnst: 1.9390 2.0978 1.8340

Red. Mass: 1.9751 2.0712 1.6166

IR Active: YES YES YES

IR Intens: 3.498 4.536 9.076

Raman Active: YES YES YES

Raman Intens: 4.806 21.999 10.341

Depolar: 0.494 0.401 0.376

Mode: 37 38 39

Frequency: 1428.99 1441.36 1458.10

Force Cnst: 1.5502 3.1965 3.1242

Red. Mass: 1.2885 2.6114 2.4941

IR Active: YES YES YES

IR Intens: 0.989 0.261 4.980

Raman Active: YES YES YES

Raman Intens: 7.115 4.081 8.817

Depolar: 0.740 0.321 0.746

Mode: 40 41 42

Frequency: 1481.47 1513.61 1519.71

Force Cnst: 1.4416 1.5630 3.3496

Red. Mass: 1.1148 1.1579 2.4616

IR Active: YES YES YES

IR Intens: 3.638 6.705 33.492

Raman Active: YES YES YES

Raman Intens: 27.080 22.444 74.613

Depolar: 0.552 0.558 0.267

Mode: 43 44 45

Frequency: 1521.21 1525.50 1615.38

Force Cnst: 2.9225 2.0337 7.7210

Red. Mass: 2.1435 1.4833 5.0219

IR Active: YES YES YES

IR Intens: 5.860 3.810 85.463

Raman Active: YES YES YES

Raman Intens: 16.394 11.646 51.737

Depolar: 0.367 0.601 0.746

**Full list of frequencies and ZPE. Note: due to the constraints (two, one torsional angle and one distance),two imaginary frequencies were found.**

**Term ZPE Enthalpy Entropy Cv % in**

**cm-1 kJ/mol kJ/mol J/mol.K J/mol.K Ground IR Int.**

**-- ---------- ------- ------- ------- ----**

**1* i 62.483 0.0000 0.0000 0.0000 0.0000 0.00 6.55**

**2* i 41.627 0.0000 0.0000 0.0000 0.0000 0.00 0.36**

**3 24.650 0.1474 2.3344 26.0209 8.3046 11.22 2.63**

**4 73.028 0.4368 2.0677 17.0289 8.2289 29.70 0.26**

**5 106.015 0.6341 1.8987 13.9770 8.1354 40.05 1.45**

**6 144.444 0.8640 1.7145 11.4814 7.9858 50.19 77.40**

**7 186.827 1.1175 1.5272 9.4519 7.7734 59.41 85.64**

**8 210.362 1.2582 1.4300 8.5375 7.6357 63.77 18.54**

**9 246.034 1.4716 1.2919 7.3588 7.4029 69.50 14.48**

**10 306.150 1.8312 1.0831 5.7866 6.9539 77.18 86.62**

**11 326.766 1.9545 1.0180 5.3388 6.7863 79.34 261.99**

**12 375.793 2.2477 0.8760 4.4185 6.3667 83.69 21.88**

**13 381.320 2.2808 0.8611 4.3259 6.3178 84.12 0.21**

**14 451.310 2.6994 0.6897 3.3129 5.6818 88.67 3.95**

**15 486.921 2.9124 0.6143 2.8938 5.3515 90.46 5.49**

**16 529.652 3.1680 0.5332 2.4601 4.9554 92.24 5.90**

**17 623.766 3.7309 0.3868 1.7177 4.1079 95.07 0.81**

**18 697.660 4.1729 0.2982 1.2923 3.4882 96.55 6.80**

**19 731.433 4.3749 0.2642 1.1336 3.2227 97.07 5.63**

**20 788.025 4.7134 0.2151 0.9090 2.8061 97.77 1.18**

**21 797.199 4.7683 0.2080 0.8769 2.7420 97.87 1.56**

**22 822.783 4.9213 0.1892 0.7931 2.5686 98.11 0.40**

**23 859.155 5.1389 0.1653 0.6870 2.3354 98.42 53.93**

**24 975.061 5.8322 0.1065 0.4327 1.6959 99.10 0.95**

**25 978.151 5.8506 0.1052 0.4274 1.6809 99.11 0.14**

**26 987.106 5.9042 0.1017 0.4123 1.6382 99.15 0.11**

**27 997.224 5.9647 0.0978 0.3958 1.5910 99.19 2.06**

**28 1069.462 6.3968 0.0738 0.2954 1.2851 99.43 5.63**

**29 1092.986 6.5375 0.0673 0.2684 1.1967 99.49 2.72**

**30 1136.477 6.7976 0.0567 0.2247 1.0469 99.58 4.17**

**31 1179.610 7.0556 0.0477 0.1882 0.9145 99.66 2.23**

**32 1216.895 7.2786 0.0411 0.1613 0.8120 99.72 4.91**

**33 1286.997 7.6980 0.0310 0.1206 0.6465 99.80 1.92**

**34 1290.818 7.7208 0.0305 0.1187 0.6385 99.80 3.50**

**35 1311.140 7.8424 0.0281 0.1091 0.5970 99.82 4.54**

**36 1387.604 8.2997 0.0205 0.0792 0.4618 99.88 9.08**

**37 1428.994 8.5473 0.0173 0.0665 0.4009 99.90 0.99**

**38 1441.362 8.6213 0.0165 0.0631 0.3842 99.90 0.26**

**39 1458.100 8.7214 0.0154 0.0588 0.3626 99.91 4.98**

**40 1481.469 8.8612 0.0139 0.0533 0.3343 99.92 3.64**

**41 1513.612 9.0534 0.0122 0.0465 0.2988 99.93 6.71**

**42 1519.714 9.0899 0.0119 0.0453 0.2925 99.93 33.49**

**43 1521.208 9.0988 0.0118 0.0450 0.2909 99.94 5.86**

**44 1525.503 9.1245 0.0116 0.0442 0.2866 99.94 3.81**

**45 1615.384 9.6621 0.0080 0.0301 0.2081 99.96 85.46**

**46 1677.482 10.0336 0.0061 0.0231 0.1663 99.97 86.28**

**47 3004.573 17.9713 0.0000 0.0001 0.0009 100.00 14.39**

**48 3052.510 18.2581 0.0000 0.0001 0.0007 100.00 21.95**

**49 3063.314 18.3227 0.0000 0.0000 0.0007 100.00 12.33**

**50 3124.133 18.6865 0.0000 0.0000 0.0005 100.00 31.03**

**51 3126.193 18.6988 0.0000 0.0000 0.0005 100.00 24.82**

**52 3173.180 18.9798 0.0000 0.0000 0.0004 100.00 12.91**

**53 3194.674 19.1084 0.0000 0.0000 0.0004 100.00 7.39**

**54 3213.060 19.2184 0.0000 0.0000 0.0004 100.00 7.28**

**55 3218.603 19.2515 0.0000 0.0000 0.0004 100.00 0.59**

**56 3792.178 22.6823 0.0000 0.0000 0.0000 100.00 9.32**

**57 3898.160 23.3162 0.0000 0.0000 0.0000 100.00 27.62**

**-- ---------- ------- ------- ------- ----**

**Total Vibrations 449.3306 20.5993 133.5123 136.3861**

**Ideal Gas 2.4789**

**Translation 3.7184 170.2934 12.4716**

**Rotation 3.7184 126.8867 12.4716**

**---------- ------- ------- -------**

**Totals 479.8457 430.6923 161.3293**

**From Figure 5H: Optimized distances (under constraints) calculated at UB3LYP/6-31G(d,p):**

**
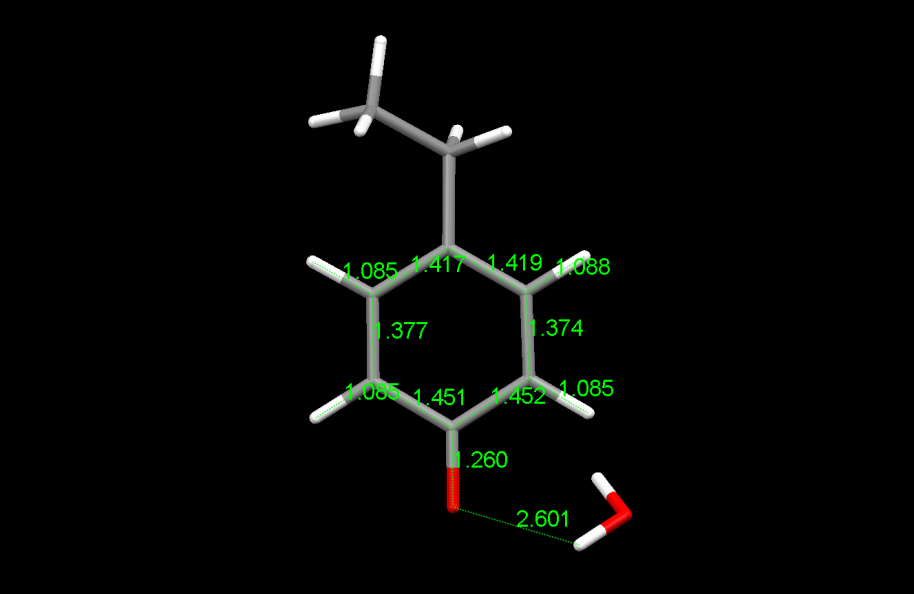
**

**From Figure 5I: The Mode: 41, corresponds to the C-O stretching vibration.**

**************************************************************************

**** ****

**** VIBRATIONAL ANALYSIS ****

**** -------------------- ****

**** ****

**** VIBRATIONAL FREQUENCIES (CM**-1) AND NORMAL MODES ****

**** FORCE CONSTANTS (mDYN/ANGSTROM) AND REDUCED MASSES (AMU) ****

**** INFRARED INTENSITIES (KM/MOL) ****

**** RAMAN SCATTERING ACTIVITIES (A**4/AMU) AND DEPOLARIZATION RATIOS ****

**** ****

**************************************************************************

**Mode: 25 26 27**

**Frequency: 1069.94 1086.64 1142.79**

**Force Cnst: 1.2697 1.1143 1.0373**

**Red. Mass: 1.8825 1.6016 1.3481**

**IR Active: YES YES YES**

**IR Intens: 6.314 5.004 8.449**

**Raman Active: YES YES YES**

**Raman Intens: 4.175 1.162 2.428**

**Depolar: 0.114 0.572 0.569**

**Mode: 28 29 30**

**Frequency: 1188.07 1219.01 1279.77**

**Force Cnst: 1.0355 1.8514 1.2144**

**Red. Mass: 1.2451 2.1147 1.2585**

**IR Active: YES YES YES**

**IR Intens: 1.974 20.307 0.543**

**Raman Active: YES YES YES**

**Raman Intens: 4.818 41.067 28.426**

**Depolar: 0.750 0.222 0.448**

**Mode: 31 32 33**

**Frequency: 1293.72 1315.61 1392.26**

**Force Cnst: 1.8358 3.0216 1.8440**

**Red. Mass: 1.8616 2.9630 1.6146**

**IR Active: YES YES YES**

**IR Intens: 3.531 1.233 6.440**

**Raman Active: YES YES YES**

**Raman Intens: 3.705 43.697 6.590**

**Depolar: 0.538 0.365 0.445**

**Mode: 34 35 36**

**Frequency: 1434.20 1442.14 1463.52**

**Force Cnst: 1.5331 2.7625 2.6581**

**Red. Mass: 1.2650 2.2544 2.1063**

**IR Active: YES YES YES**

**IR Intens: 0.938 2.087 9.729**

**Raman Active: YES YES YES**

**Raman Intens: 5.963 5.051 9.208**

**Depolar: 0.642 0.463 0.638**

**Mode: 37 38 39**

**Frequency: 1469.20 1513.79 1520.50**

**Force Cnst: 1.5961 1.4203 3.9798**

**Red. Mass: 1.2550 1.0519 2.9217**

**IR Active: YES YES YES**

**IR Intens: 6.862 5.940 9.334**

**Raman Active: YES YES YES**

**Raman Intens: 52.415 13.723 3.334**

**Depolar: 0.448 0.748 0.679**

**Mode: 40 41 42**

**Frequency: 1525.00 1554.09 1631.99**

**Force Cnst: 1.6024 7.4329 8.2966**

**Red. Mass: 1.1695 5.2234 5.2871**

**IR Active: YES YES YES**

**IR Intens: 13.455 258.876 52.286**

**Raman Active: YES YES YES**

**Raman Intens: 31.272 313.884 24.016**

**Depolar: 0.498 0.326 0.202**

**Figure S2.** Calculated IR spectrum (See Figure 5A in the main article text)


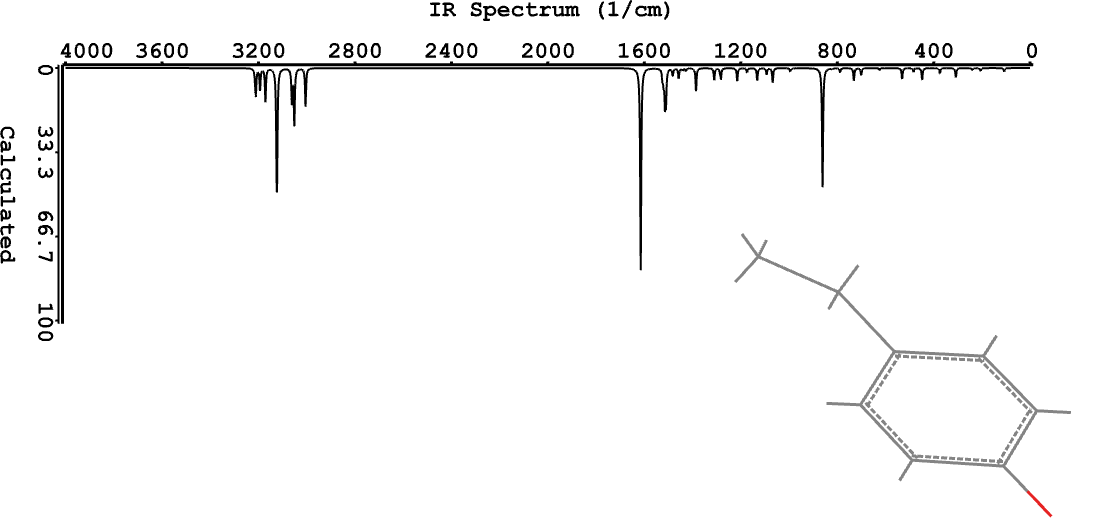


**Figure S3.** Calculated IR spectrum (see Figure 5B in the main article text)


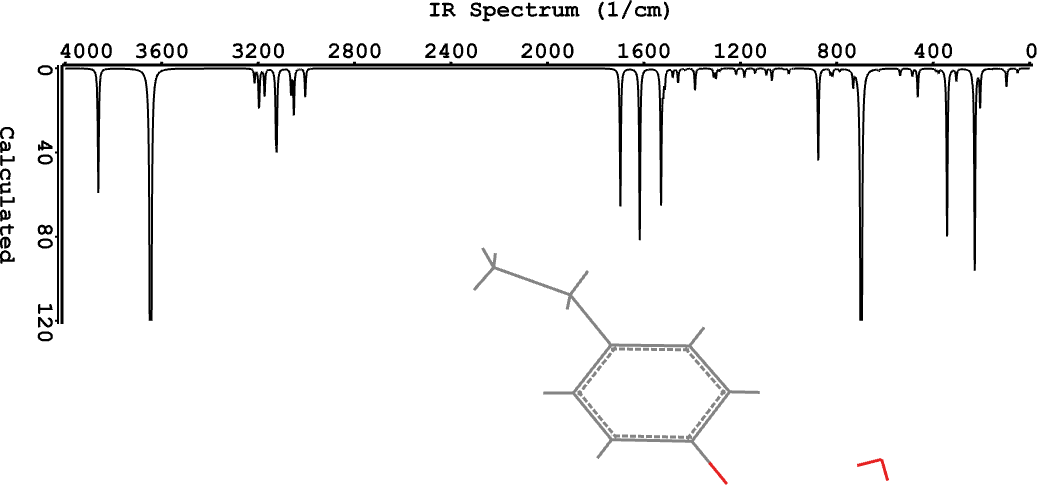


**Figure S4.** Calculated IR spectrum (see Figure 5C in the main article text)


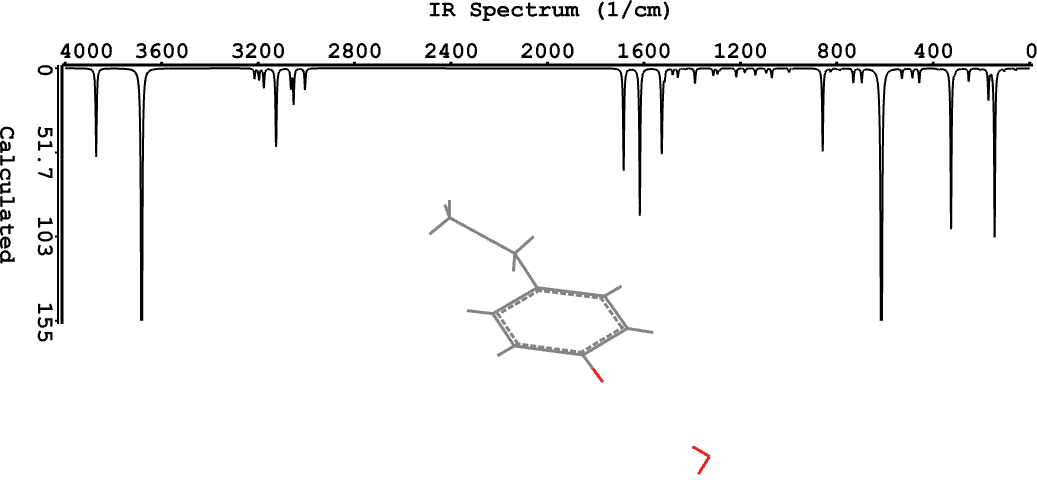


**Figure S5.** Calculated IR spectrum (see Figure 5D in the main article text)


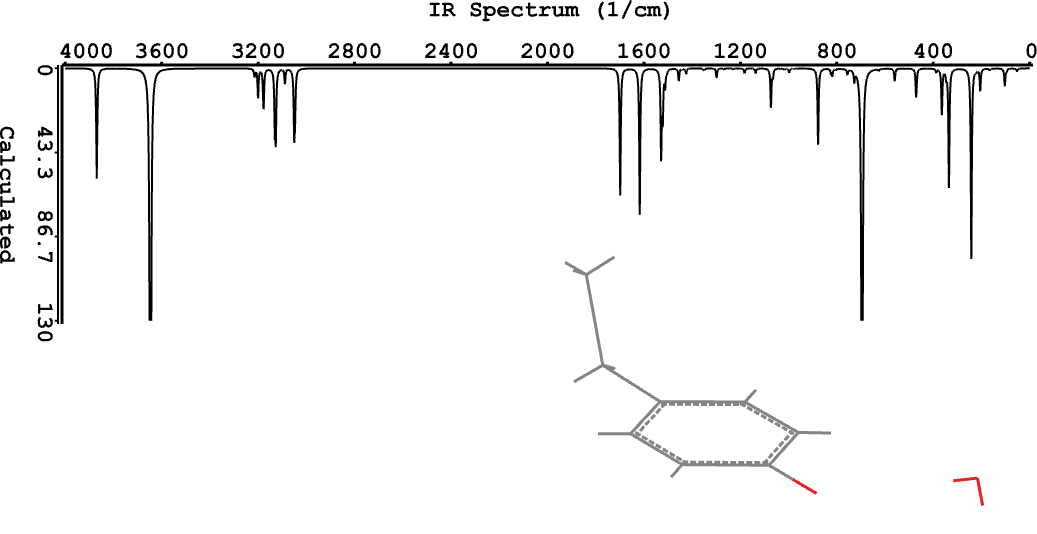


**Figure S6.** Calculated IR spectrum (see Figure 5E in the main article text)


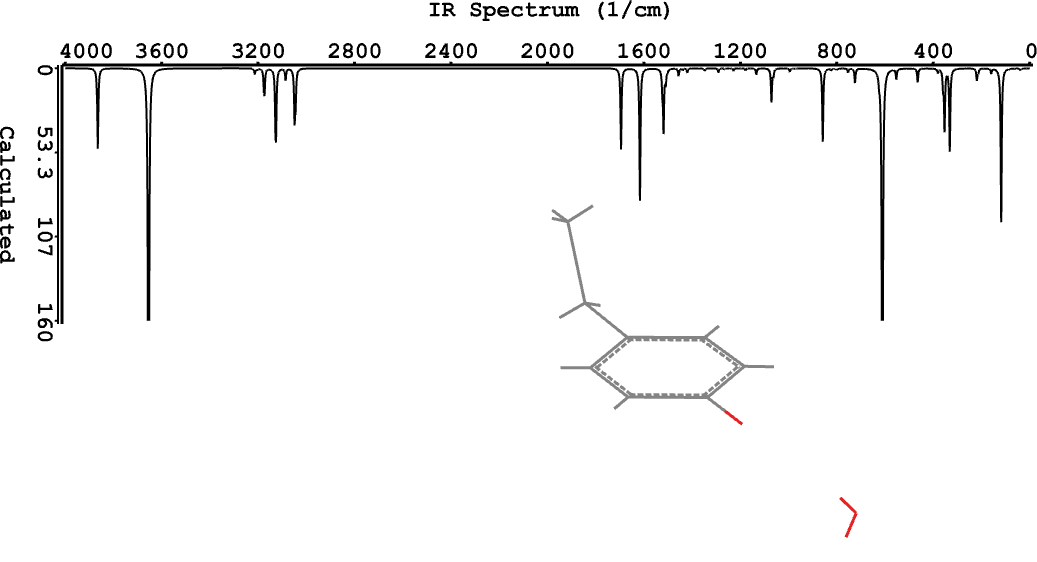


**Figure S7.** Calculated IR spectrum (see Figure 5F in the main article text)


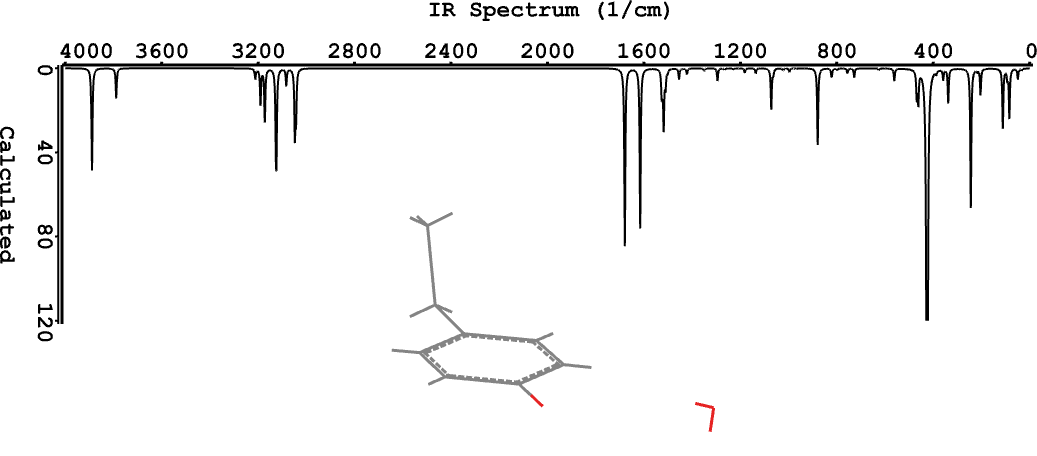


**Figure S8.** Calculated IR spectrum (see Figure 5G in the main article text)


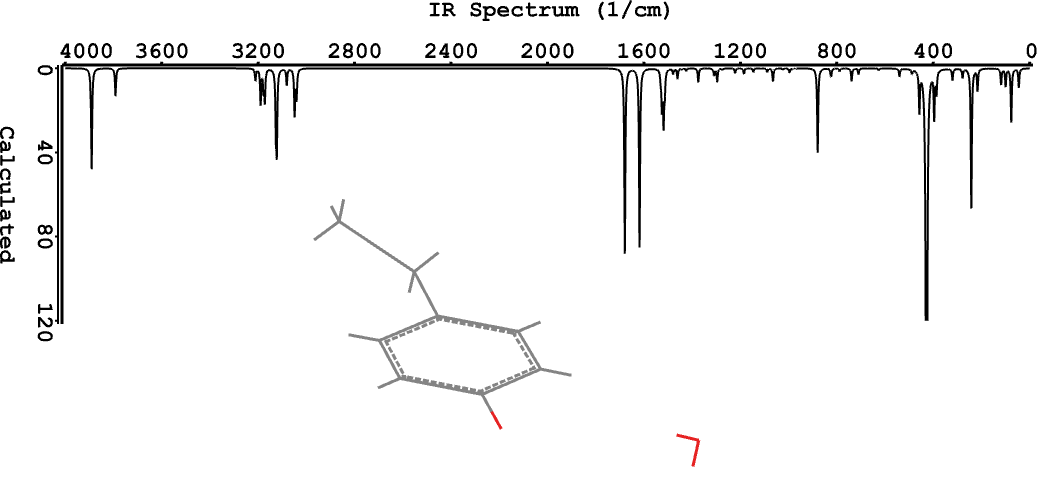


**Figure S9.** Calculated IR spectrum (see Figure 5H in the main article text)


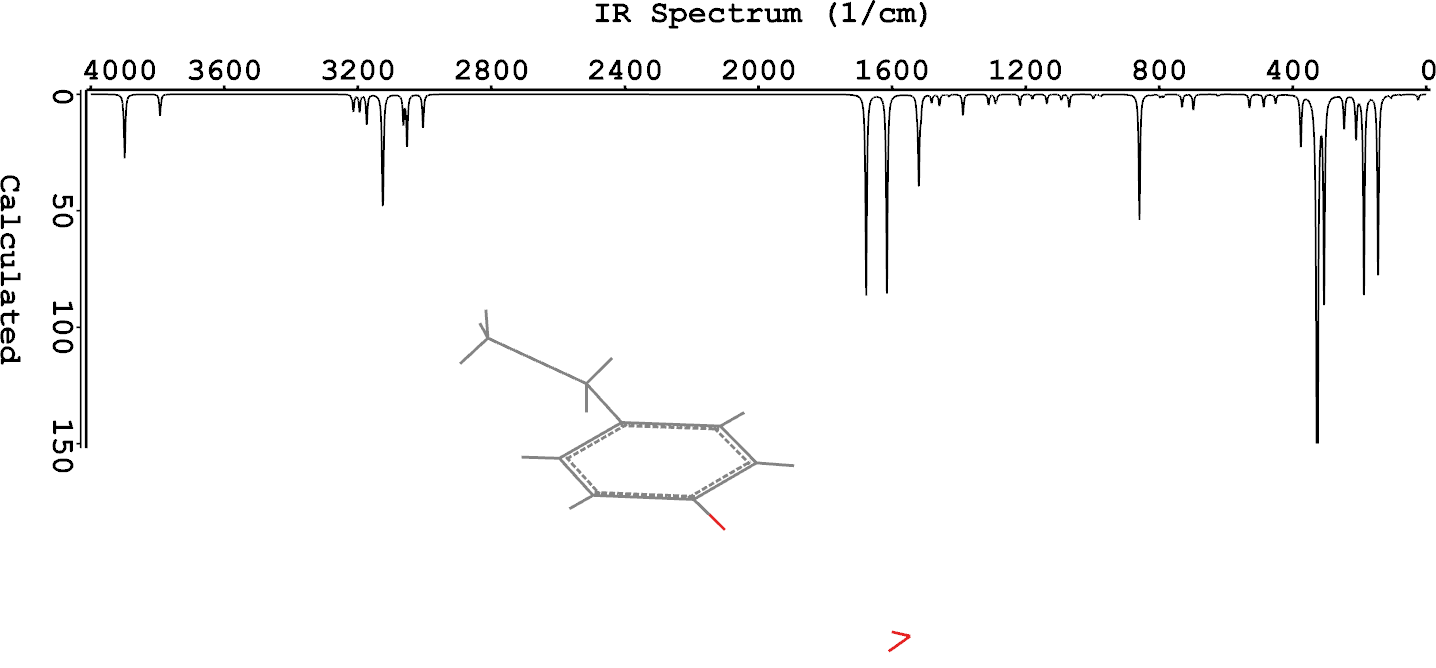


**Figure S10.** Calculated IR spectrum of the ethylphenoxyl radical under constraint angle (θ = 30°) at UB3LYP/6-31G(d,p) with Euler-Maclaurin-Lebedev (EML) grid (70,302), obtained after placing a positive charge (Li^+^) at 2.60Å on the phenoxyl plane. The mode 41 corresponds to the C-O (ν_7a_) stretching vibration.


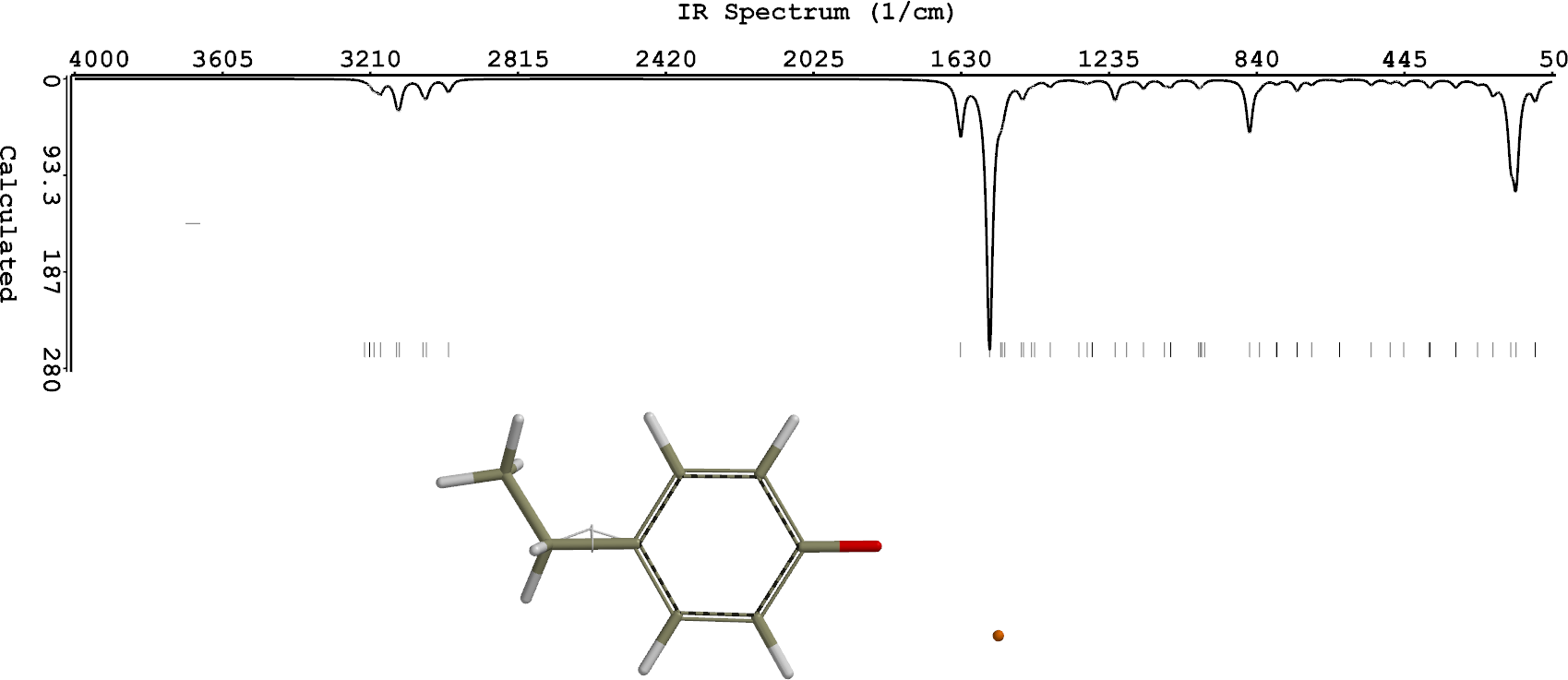


**Figure S11.** Calculated IR spectrum of the ethylphenoxyl radical, neutral form (UB3LYP/6-31G(d,p)) after optimization without constraints. The Mode: 36, corresponds to the C-O stretching vibration (ν_7a_, Wilson mode).


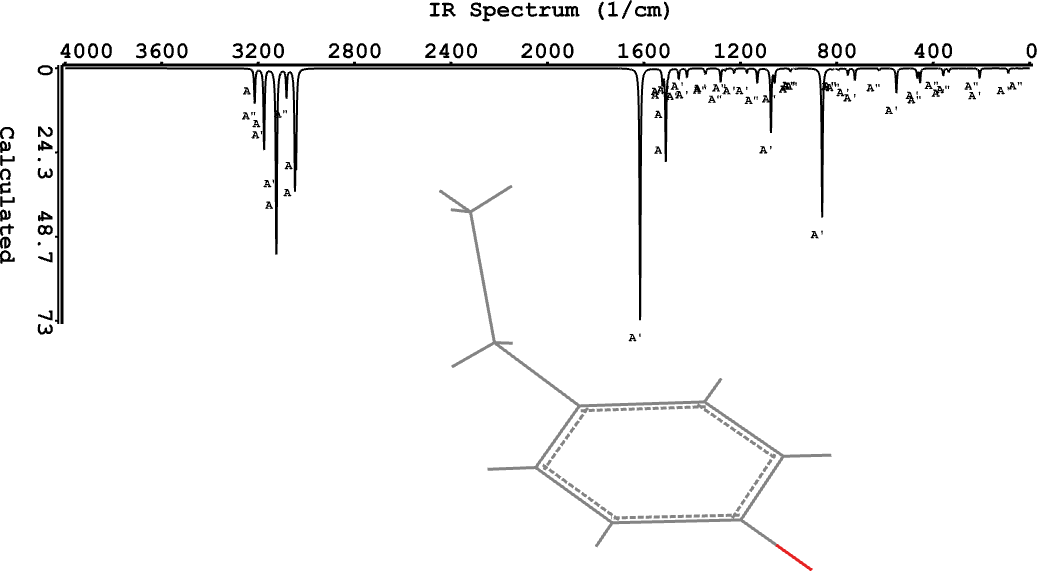


From Figure S11

**********************************************************************

** **

** VIBRATIONAL ANALYSIS **

** -------------------- **

** **

** VIBRATIONAL FREQUENCIES (CM**-1) AND NORMAL MODES **

** FORCE CONSTANTS (mDYN/ANGSTROM) AND REDUCED MASSES (AMU) **

** INFRARED INTENSITIES (KM/MOL) **

** RAMAN SCATTERING ACTIVITIES (A**4/AMU) AND DEPOLARIZATION RATIOS **

** **

**********************************************************************

Mode: 22 23 24

Frequency: 1057.95 1073.36 1129.95

Force Cnst: 0.9739 1.2467 0.9560

Red. Mass: 1.4768 1.8367 1.2708

IR Active: YES YES YES

IR Intens: 3.900 18.550 4.294

Raman Active: YES YES YES

Raman Intens: 2.089 16.013 0.521

Depolar: 0.750 0.154 0.750

Mode: 25 26 27

Frequency: 1173.19 1226.05 1263.82

Force Cnst: 0.9642 2.2216 1.4323

Red. Mass: 1.1890 2.5085 1.5220

IR Active: YES YES YES

IR Intens: 1.418 1.468 0.723

Raman Active: YES YES YES

Raman Intens: 4.993 17.702 2.135

Depolar: 0.574 0.168 0.750

Mode: 28 29 30

Frequency: 1281.87 1344.24 1347.12

Force Cnst: 1.9974 2.0963 1.6189

Red. Mass: 2.0632 1.9690 1.5141

IR Active: YES YES YES

IR Intens: 3.993 1.375 0.781

Raman Active: YES YES YES

Raman Intens: 2.463 8.275 50.080

Depolar: 0.750 0.750 0.425

Mode: 31 32 33

Frequency: 1421.76 1441.17 1455.98

Force Cnst: 1.4494 3.3628 3.4298

Red. Mass: 1.2170 2.7480 2.7461

IR Active: YES YES YES

IR Intens: 2.640 0.140 3.136

Raman Active: YES YES YES

Raman Intens: 6.060 4.616 12.434

Depolar: 0.618 0.348 0.750

Mode: 34 35 36

Frequency: 1499.79 1509.16 1509.51

Force Cnst: 1.4827 1.3997 4.3165

Red. Mass: 1.1188 1.0431 3.2152

IR Active: YES YES YES

IR Intens: 1.230 8.307 18.622

Raman Active: YES YES YES

Raman Intens: 24.529 19.597 71.697

Depolar: 0.704 0.750 0.251

Mode: 37 38 39

Frequency: 1522.78 1523.44 1615.99

Force Cnst: 1.4956 6.3803 7.9326

Red. Mass: 1.0947 4.6660 5.1557

IR Active: YES YES YES

IR Intens: 2.716 1.665 72.843

Raman Active: YES YES YES

Raman Intens: 5.901 0.576 61.466

Depolar: 0.437 0.750 0.731

**PART III:** Single point calculations on the ethylphenoxyl radical under constraint angle (θ = 30°) at UB3LYP/6-31G(d,p) with Euler-Maclaurin-Lebedev (EML) grid (70,302), neutral form, including solvation model [SM8](3) performed in water, ethanol, diethylether. In blue the value of the spin-density for the phenoxyl oxygen and in red the C(4) atom.


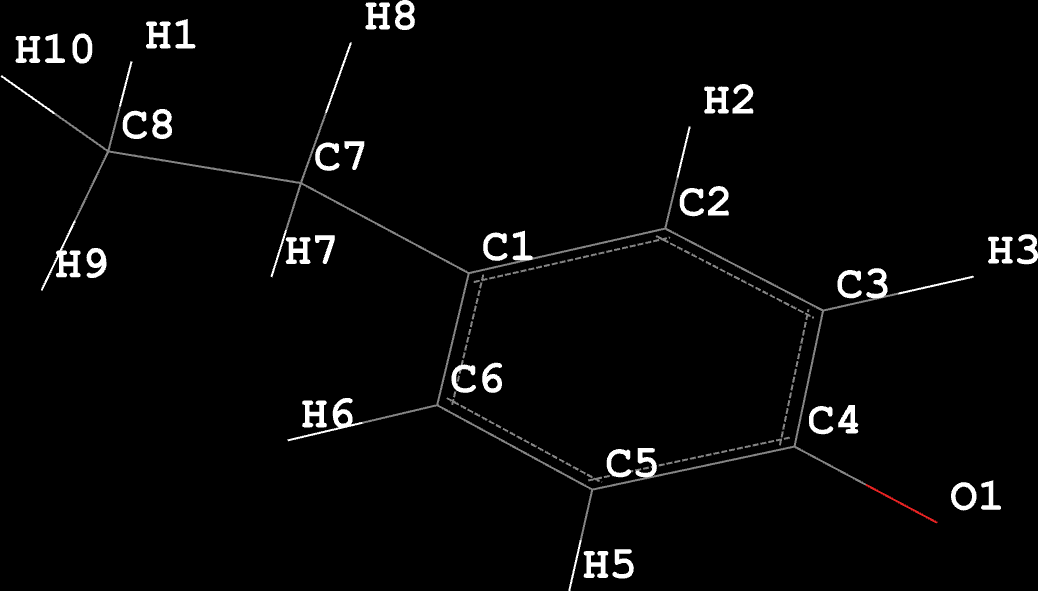


**Figure S10**

**Solvation: water [SM8]**

Free Energy of Solvation : -20.8669104 kJ/mol

SCF total energy: -385.4808211 hartrees

Natural Atomic Populations and Charges (labels given in Figure S10)

Alpha spin Beta spin Spin

Atom Occupancy Occupancy Density Charge

------ ----------- ----------- ----------- ------------

C1 3.145491 2.785940 0.359551 0.068569

C4 2.800878 2.816941 -0.016062 0.382181

C2 3.067061 3.170648 -0.103587 -0.237708

C6 3.069801 3.175886 -0.106086 -0.245687

C5 3.239782 2.981287 0.258494 -0.221069

C3 3.235043 2.991497 0.243546 -0.226540

H2 0.374895 0.372161 0.002734 0.252944

H6 0.374407 0.371628 0.002779 0.253965

H5 0.372977 0.380742 -0.007766 0.246281

H3 0.372861 0.380228 -0.007367 0.246910

C7 3.239033 3.252365 -0.013332 -0.491398

H7 0.376108 0.353465 0.022643 0.270427

H8 0.374821 0.368894 0.005927 0.256284

C8 3.347575 3.342491 0.005084 -0.690067

H1 0.380666 0.380373 0.000293 0.238960

H9 0.383613 0.383737 -0.000123 0.232650

H10 0.376781 0.376595 0.000186 0.246624

O1 4.468206 4.115120 0.353086 -0.583326

Ground-State Mulliken Net Atomic Charges

Atom Charge (a.u.) Spin (a.u.)

--------------------------------------------------------

1 C 0.143862 0.413564

2 C 0.087941 -0.038055

3 C -0.076153 -0.144486

4 C -0.071043 -0.148537

5 C -0.056978 0.294969

6 C -0.056280 0.278115

7 H 0.124301 0.004629

8 H 0.124415 0.004760

9 H 0.096609 -0.013119

10 H 0.096366 -0.012406

11 C -0.235827 -0.029152

12 H 0.148796 0.026073

13 H 0.128890 0.007258

14 C -0.328452 0.005620

15 H 0.118436 0.001065

16 H 0.114474 -0.000244

17 H 0.111727 0.000697

18 O -0.471086 0.349250

--------------------------------------------------------

Sum of atomic charges = 0.000000

Sum of spin charges = 1.000000

**Solvation: ethanol [SM8]**

Free Energy of Solvation : -34.1313559 kJ/mol

SCF total energy: -385.4858733 hartrees

Natural Atomic Populations and Charges (labels given in Figure S10)

Alpha spin Beta spin Spin

Atom Occupancy Occupancy Density Charge

------ ----------- ----------- ----------- ------------

C1 3.146059 2.789027 0.357032 0.064914

C4 2.798183 2.822651 -0.024468 0.379166

C2 3.067095 3.172555 -0.105460 -0.239649

C6 3.069831 3.177664 -0.107833 -0.247495

C5 3.240776 2.980479 0.260297 -0.221255

C3 3.236054 2.990523 0.245531 -0.226577

H2 0.375420 0.372622 0.002798 0.251957

H6 0.374884 0.372046 0.002838 0.253069

H5 0.372922 0.380743 -0.007820 0.246335

H3 0.372804 0.380229 -0.007426 0.246967

C7 3.238833 3.252261 -0.013428 -0.491094

H7 0.376398 0.354028 0.022370 0.269574

H8 0.375062 0.369210 0.005852 0.255727

C8 3.347523 3.342493 0.005030 -0.690015

H1 0.380751 0.380468 0.000284 0.238781

H9 0.383679 0.383806 -0.000127 0.232515

H10 0.376856 0.376676 0.000181 0.246468

O1 4.466869 4.102519 0.364350 -0.569388

Ground-State Mulliken Net Atomic Charges

Atom Charge (a.u.) Spin (a.u.)

--------------------------------------------------------

1 C 0.141617 0.411122

2 C 0.140618 -0.047880

3 C -0.079167 -0.146673

4 C -0.073578 -0.150554

5 C -0.073861 0.297447

6 C -0.072531 0.280819

7 H 0.121378 0.004732

8 H 0.121731 0.004857

9 H 0.096052 -0.013244

10 H 0.095858 -0.012536

11 C -0.236499 -0.029083

12 H 0.147644 0.025712

13 H 0.127972 0.007155

14 C -0.328371 0.005556

15 H 0.118194 0.001048

16 H 0.114284 -0.000250

17 H 0.111710 0.000684

18 O -0.473053 0.361089

--------------------------------------------------------

Sum of atomic charges = 0.000000

Sum of spin charges = 1.000000

**Solvation: diethylether [SM8]**

Free Energy of Solvation : -32.0613020 kJ/mol

SCF total energy: -385.4850849 hartrees

Natural Atomic Populations and Charges (labels given in Figure S10)

Alpha spin Beta spin Spin

Atom Occupancy Occupancy Density Charge

------ ----------- ----------- ----------- ------------

C1 3.148613 2.799697 0.348916 0.051690

C4 2.788415 2.840129 -0.051714 0.371456

C2 3.067472 3.179022 -0.111550 -0.246494

C6 3.070166 3.183692 -0.113526 -0.253858

C5 3.243768 2.977486 0.266282 -0.221254

C3 3.239061 2.986962 0.252099 -0.226023

H2 0.377411 0.374408 0.003003 0.248180

H6 0.376688 0.373657 0.003031 0.249655

H5 0.372535 0.380534 -0.008000 0.246931

H3 0.372406 0.380026 -0.007620 0.247568

C7 3.238296 3.252074 -0.013779 -0.490370

H7 0.377595 0.356118 0.021476 0.266287

H8 0.376048 0.370442 0.005605 0.253510

C8 3.347408 3.342552 0.004856 -0.689960

H1 0.380962 0.380711 0.000252 0.238327

H9 0.383892 0.384033 -0.000141 0.232075

H10 0.377132 0.376971 0.000161 0.245897

O1 4.462133 4.061486 0.400647 -0.523619

Ground-State Mulliken Net Atomic Charges

Atom Charge (a.u.) Spin (a.u.)

--------------------------------------------------------

1 C 0.134144 0.403273

2 C 0.285794 -0.080451

3 C -0.097117 -0.153833

4 C -0.089905 -0.157171

5 C -0.106562 0.305968

6 C -0.103176 0.290049

7 H 0.110099 0.005063

8 H 0.111468 0.005167

9 H 0.098390 -0.013636

10 H 0.098288 -0.012949

11 C -0.240939 -0.028880

12 H 0.143089 0.024512

13 H 0.124162 0.006808

14 C -0.328309 0.005346

15 H 0.117853 0.000991

16 H 0.113699 -0.000270

17 H 0.111673 0.000642

18 O -0.482651 0.399372

--------------------------------------------------------

Sum of atomic charges = 0.000000

Sum of spin charges = 1.000000
